# Recovery Kinetics of Knee Flexor and Extensor Strength after a Football Match

**DOI:** 10.1371/journal.pone.0128072

**Published:** 2015-06-04

**Authors:** Dimitrios Draganidis, Athanasios Chatzinikolaou, Alexandra Avloniti, José C. Barbero-Álvarez, Magni Mohr, Paraskevi Malliou, Vassilios Gourgoulis, Chariklia K. Deli, Ioannis I. Douroudos, Konstantinos Margonis, Asimenia Gioftsidou, Andreas D. Fouris, Athanasios Z. Jamurtas, Yiannis Koutedakis, Ioannis G. Fatouros

**Affiliations:** 1 School of Physical Education and Sports Science, Democritus University of Thrace, Komotini, Greece; 2 University of Granada, Campus of Melilla, Melilla, Spain; 3 Faculty of Natural and Health Science, University of the Faroe Islands, Tórshavn, Faroe Islands; 4 Centre of Health and Human Performance, Department of Food and Nutrition, and Sport Sciences, University of Gothenburg, Gothenburg, Sweden; 5 School of Physical Education and Sports Science, University of Thessaly, Trikala, Greece; 6 Institute of Human Performance and Rehabilitation, Centre for Research and Technology, Thessaly (CERETETH), Trikala, Greece; 7 School of Sports, Performing Arts and Leisure, University of Wolverhampton, Wolverhampton, United Kingdom; Semmelweis University, HUNGARY

## Abstract

We examined the temporal changes of isokinetic strength performance of knee flexor (KF) and extensor (KE) strength after a football match. Players were randomly assigned to a control (N = 14, participated only in measurements and practices) or an experimental group (N = 20, participated also in a football match). Participants trained daily during the two days after the match. Match and training overload was monitored with GPS devices. Venous blood was sampled and muscle damage was assessed pre-match, post-match and at 12h, 36h and 60h post-match. Isometric strength as well as eccentric and concentric peak torque of knee flexors and extensors in both limbs (dominant and non-dominant) were measured on an isokinetic dynamometer at baseline and at 12h, 36h and 60h after the match. Functional (KF_ecc_/KE_con_) and conventional (KF_con_/KE_con_) ratios were then calculated. Only eccentric peak torque of knee flexors declined at 60h after the match in the control group. In the experimental group: a) isometric strength of knee extensors and knee flexors declined (P<0.05) at 12h (both limbs) and 36h (dominant limb only), b) eccentric and concentric peak torque of knee extensors and flexors declined (P<0.05) in both limbs for 36h at 60°/s and for 60h at 180°/s with eccentric peak torque of knee flexors demonstrating a greater (P<0.05) reduction than concentric peak torque, c) strength deterioration was greater (P<0.05) at 180°/s and in dominant limb, d) the functional ratio was more sensitive to match-induced fatigue demonstrating a more prolonged decline. Discriminant and regression analysis revealed that strength deterioration and recovery may be related to the amount of eccentric actions performed during the match and athletes' football-specific conditioning. Our data suggest that recovery kinetics of knee flexor and extensor strength after a football match demonstrate strength, limb and velocity specificity and may depend on match physical overload and players' physical conditioning level.

## Introduction

During Association football (soccer) competition, football players cover a distance of 9–13 km at high intensity including >200 high-intensity runs that require demanding changes in direction [1] with forceful accelerations/decelerations [2] causing fatigue during and at the end of a game [3]. Daily in-season training protocols incorporate a similar activity profile of lower volume compared to match-play [4]. The recovery process from a game is slow compared to continuous sports of similar duration [5], which may be associated with the specific movement pattern of the game provoking muscle injuries, inflammation and impaired recovery [6]. As professional players may participate in >70 matches/season, interspersed with 3 to 6-day training sessions over the course of a ~10 month long season, accumulated fatigue may deteriorate performance and increase inflammation that may predispose athletes to injuries [7].

Injuries may occur during practice and match-play with hamstring tears and anterior cruciate ligament (ACL) ruptures being more common [8]. Imbalances between the strength of knee extensors (KE) and flexors (KF), poor eccentric strength of KF and bilateral strength asymmetries may represent serious injury risk factors in professional and semiprofessional players [9] although a recent systematic review concluded that evidence for the risk factor muscle imbalance are still inconclusive [10]. During forceful knee extensions when running and kicking, hamstrings contract eccentrically to counteract anterior shear forces and decelerate tibia's forward movement and internal rotation during the later part of a forward swing phase [8]. It has been documented that isokinetic evaluation of lower limb strength and use of KF:KE ratios to evaluate potential asymmetries is a valuable screening tool for injury risk and development of injury prevention strategies [11]. Indeed, abnormal conventional (KF peak concentric torque to KE peak concentric torque, KF_con_/KE-_con_) and functional (KF peak eccentric torque to the KE peak concentric torque, KF_ecc_/KE_con_) ratios have been associated with hamstring/ACL injury risk [12]. Fatigue induced by simulated intense football activity significantly altered both ratios due to a greater loss of KF strength [13–15] rendering KF more susceptible to stretch injury [9]. However, the use of KF_con_/KE_con_ has been questioned since opposing muscles cannot contract concentrically simultaneously whereas KF_ecc_/KE_con_ evaluates muscle actions taking place simultaneously [12]. Reduced KF_ecc_/KE_con_ indicates suboptimal KF strength to decelerate the tibia at the end of a forceful eccentric contraction predisposing the musculo-tendinous unit to tearing [9, 11, 12]. Consequently, deceleration is limited and KF, in order to maintain limb's forward momentum, must produce a greater eccentric contraction which may then cause their tearing [9, 11, 12].

The occurrence of acceleration/deceleration motions during running, sprinting, tackling, turning, changing pace, physical contact with opposition, jumping and changes in direction have been associated with muscle damage induced by football matches and their frequency may affect post-match recovery kinetics [6, 16]. These activities incorporate a strong eccentric component which is associated with the onset of muscle damage [17] eliciting muscular pain, acute inflammatory response and performance deterioration for as long as 1–5 days after a match [18, 19]. KF may be more susceptible to injury during the rapid transition from their eccentric to concentric contraction, when they act to extend the hip [20]. Evidence suggest that at the end of each half hamstrings tears and ACL ruptures are increased because of KF reduced ability to contract eccentrically due to accumulated fatigue [20] suggesting that KF_ecc_/KE_con_ fluctuations may be a better predictor of football injuries. However, no information exists about the temporal changes of KF_ecc_/KE_con_ and KF_con_/KE_con_ in response to football match-play in competitive players. Most previous studies utilized non-specific, intermittent running protocols on a treadmill or football field to simulate football activity that lack football-specific movement patterns. Thus, the present study aimed to i) examine the temporal effect of football match-induced fatigue on changes in KF and KE strength performance at slow vs. fast contraction velocities, ii) investigate whether the conventional or the functional ratio is more affected by a football game, iii) determine whether KF and KE strength performance fluctuations demonstrate a different response pattern in dominant vs. non-dominant limb, iv) determine factors that may affect strength decline during the inflammatory phase after a football game and v) determine factors that may affect kinetics of strength recovery during the recovery phase of the post-game period. Consequently, our initial null research hypotheses state that: i) strength performance fluctuations of KE and KF following a football game will be related to knee velocity, ii) the conventional and the functional ratios will respond differently to a football game, iii) dominant and non-dominant limbs will demonstrate a different response pattern of KE and KF responses, iv) KE and KF strength decline and recovery following a football game will be associated with players' conditioning status and v) the rate of strength recovery of KF and KE after the game will be related to high-intensity activities performed by the players during the game.

## Materials and Methods

### Ethics statement

Participants signed a consent form after they were informed of all risks, discomforts and benefits involved in the study. Procedures were in agreement with the 1975 Declaration of Helsinki, as revised in 2000, and approval was granted by the institutional ethics committee of the Department of Physical Education and Sports Sciences of the University of Thessaly.

### Participants

A preliminary power analysis indicated that 12–16 participants/group were required to detect statistically meaningful treatment effects between consecutive measurements after a football match (α = 0.90). Accordingly, 34 healthy male semi-professional players (12 defenders, 14 midfielders, eight attackers) participated voluntarily in this study. Selection criteria included: a) participation in football competition for ≥5 years, b) no recent musculoskeletal injuries and/or other illnesses, c) abstinence from ergogenic supplements, smoking and medications (≥6 months), d) participation in ≥6 training sessions/week and ≥1 match/week, e) knee flexor/extensor ratio within the normal range, i.e. KE_con_/KF_con_ 0.61–0.70 [21, 22] and KE_ecc_/KF_con_ 0.68–0.98 [21], and f) a strength difference between dominant limb and non-dominant limb ≤10% since differences ≥15% may predispose to knee injuries [23]. Participants’ characteristics are shown in Table 1.

**Table 1:** Participants’ characteristics.

	Control Group	Experimental Group
	(N = 20)	(N = 14)
**Age (years)**	22.6 ± 1.5	23.1 ± 2.7
**Time at competitive level (years)**	10.6 ± 1.4	10.4 ± 0.9
**Body mass (kg)**	74.3 ± 4.8	75.4 ± 6.1
**Height (m)**	1.79 ± 0.04	1.81 ± 0.06
**BMI (kg/m** ^**2**^ **)**	23.2 ± 0.4	22.9 ± 0.5
**Body fat (%)**	7.0 ± 1.0	7.3 ± 1.6
**VO**_**2max**_ **(mL/kg/min)**	58.9 ± 5.1	59.5 ± 5.5
**HR**_**max**_ **(beats/sec)** ^**1**^	198.1 ± 5.3	198.9 ± 6.1
**Yo-Yo IR2 (m)**	1394.7 ± 208.3	1330.8 ± 148.5

BMI, body mass index; VO_2max_, maximal oxygen consumption; HR, heart rate; HRmax, maximal heart rate; Yo-Yo IR2, Yo-Yo intermittent recovery test-level 2; ^1^recorded during the graded exercise test used for the assessment of VO_2max_.

### Experimental design

**Fig 1:**
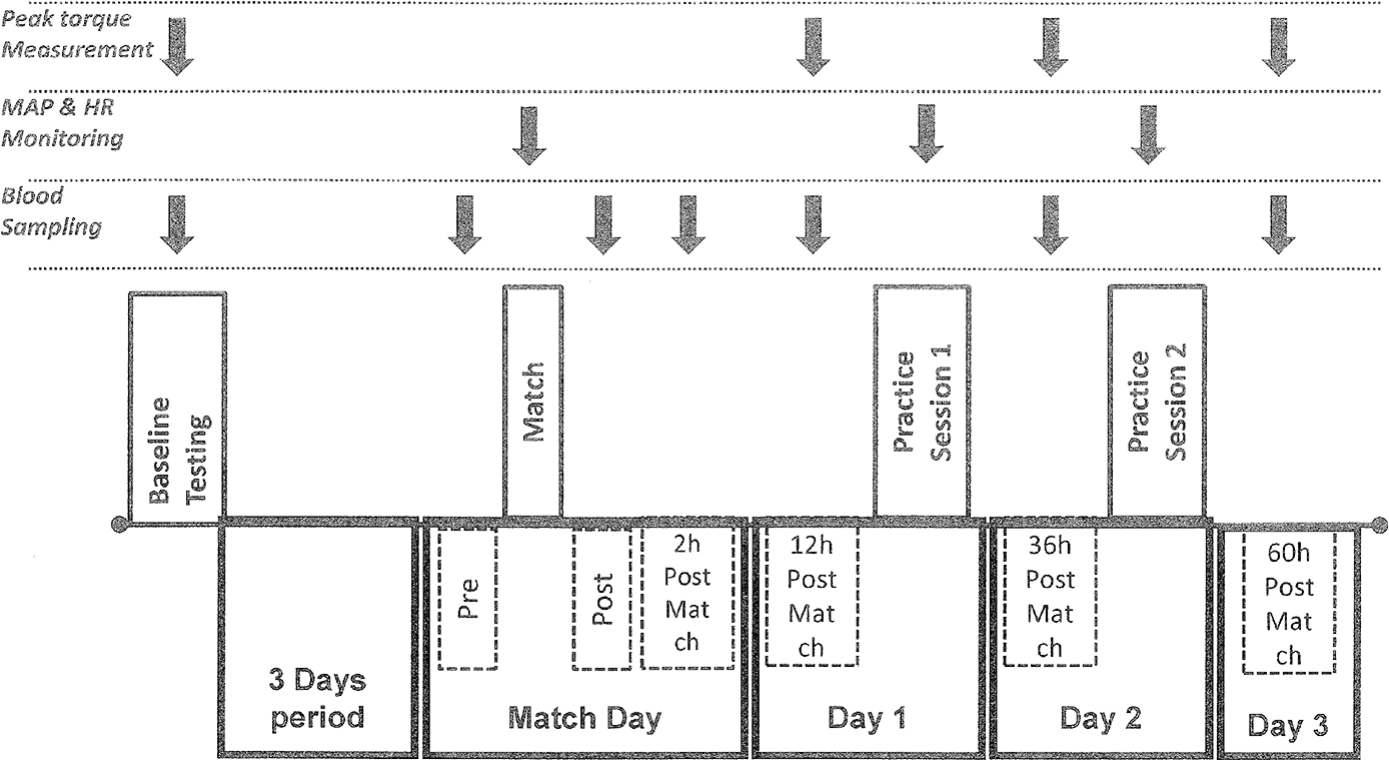
The experimental flowchart. MAP, measurement of activity (football) profile; HR, heart rate; Pre, measurements before the match; Post, measurements immediately after the match; black arrow, indicates time of measuement.

A two-group, repeated measures design was used. The study was conducted one week after completion of the in-season period while players were still accustomed to vigorous training and match loads. Three days after baseline testing, participants played a 90-min match according to official regulations. Athletes were randomly assigned to either a) a control group (C: participated only in daily training and measurements, N = 14) to account for the day-to-day variation of dependent variables or b) an experimental group (EG, participated in a match, N = 20). Participants in EG were randomly assigned to two different teams (each team consisted of a goalkeeper, four defenders, four mid-fielders, two forwards). Two matches were performed and 10 field players were monitored each time. Players participated in the entire match. On match day (18.00–20.00), athletes consumed a light standardized meal as described [19]. During the match, participants were allowed to drink only water *ad libitum*. Athletes' activity profile and heart rate was monitored throughout the match. Blood sampling and measurement of muscle damage markers were performed before the match, 3–4 min post-match (lactate measurement only) and 2h, 12h, 36h and 60h post-match. KF and KE peak torque was measured at baseline and 12h, 36h and 60h after the match. Since subjects in C did not participate in the experimental game, mean heart rate and peak heart rate values as well as post-game lactate values were not recorded and are not reported. During the post-match period (two days), athletes of both groups participated in one practice session/day (conducted by the investigators) simulating in-season training sessions of corresponding days (Table 2). To ensure that physical load of training was comparable among groups, athletes’ activity and heart rate was continuously monitored (Table 3). During practice days, all measurements and blood sampling were performed before practice (in the morning). A visual timeline of the experimental design is provided (Fig 1).

**Table 2:** The contents and duration of daily training sessions performed during the post-match period.

Practices	Contents	Duration
Recovery Day 1—Practice 1	Game group (Recovery Training)	50 min
	General warm-up (Slow jog, static/dynamic stretches)	
	Specific warm-up (Shuttle runs, small passing, dribbling drills,	
	passing combination drills, mobility exercises, coordination drills)	
	Moderate-intensity endurance running	
	Cool down	
Recovery Day 2—Practice 2	General warm-up: jogging, static and dynamic stretching exercises	70 min
	Specific warm-up: shuttle running, passing and dribbling drills,	
	mobility exercises, coordination drills	
	Technical training: passing, dribbling and shooting combination drills	
	and agility drills	
	Tactical/technical training: tactical half-court sided games, set plays	
	Cool down: light running, static and dynamic stretching exercises, core	

**Table 3:** Physical activity data obtained during practice by GPS and heart rate monitoring.

	Practice 1	Practice 2
**Mean Heart Rate (beats/min)**		
**C**	125.2 ± 7.0	140.4 ± 7.4^1^
**EG**	129.5 ± 6.5	142.1 ± 5.9^1^
**Maximal Heart Rate (beats/min)**		
**C**	179.7 ± 6.9	188.1 ± 6.1^1^
**EG**	176.3 ± 7.4	186.6 ± 4.8^1^
**Total distance (m)**		
**C**	3,275.4 ± 229.8	6,234.8 ± 86.3^1^
**EG**	3,154.7 ± 109.9	6,095.6 ± 85.6^1^
**Distance covered at 0–7.2 km/h (m)**		
**C**	2,456.2 ± 233.5	3,179.8 ± 138.4^1^
**EG**	2,302.2 ± 103.1	3,230.7 ± 101.8^1^
**Distance covered at 7.3–14.4 km/h (m)**		
**C**	556.7 ± 5.5	2,057.4 ± 120.0^1^
**EG**	599.4 ± 13.1	1,889.6 ± 60.6^1^
**Distance covered at 14.5–19.8 km/h (m)**		
**C**	163.7 ± 3.2	623.4 ± 3.6^1^
**EG**	182.9 ± 5.4	670.5 ± 9.5^1^
**Distance covered at 19.9–25.2 km/h (m)**		
**C**	68.8 ± 3.1	244.8 ± 4.0^1^
**EG**	47.3 ± 5.6	221.4 ± 3.3^1^
**Distance covered at >25.2 km/h (m)**		
**C**	30.0 ± 3.1	129.5 ± 2.5^1^
**EG**	22.7 ± 1.7	83.5 ± 2.6^1^

^1^ Denotes a significant difference with practice 1 at P<0.05; ^2^ Denotes a significant difference with practice 2 at P<0.05; GPS, global positioning system; C, control group; EG, experimental group.

### Assessment of anthropometric profile, performance and muscle damage markers

Participants had their body mass, height and percent body fat measured as described [24]. Maximal oxygen consumption (VO_2max_) and Yo-Yo intermittent recovery (Yo-Yo IR2) testing were performed on separate days as described [25, 26]. Muscle soreness (MS) of KE and KF was measured after performing three repetitions of a full squat as described [17].

### Measurement of football physical activity and assessment of isokinetic and isometric muscle function

Physical activity during match and practices was monitored using GPS Technology (SPI Elite, GPSports Systems, Australia). Participants wore a GPS device (15 Hz) equipped with a triaxial accelerometer (100 Hz). Devices were fitted on the upper back using an adjustable neoprene harness and were turned on 45 minutes before use (at least four satellites were detected). The system's validity and reliability is 1.7% [27] and have been applied in football match-play [28] and training [29]. Data recorded were exported to software (Team AMS software V 2.1.0.6, GPSports) for subsequent analyses. Match- and practice-related activities were classified as: standing/walking (0.5–7.2 km/h), jogging (7.3–14.4 km/h), running (14.5–19.8 km/h), high-intensity running (19.9–25.2 km/h) and sprinting (>25.2 km/h). Participants' number of high-intensity accelerations and decelerations (1–2 m/s^2^, 2–3 m/s^2^, > 3 m/s^2^) and total distance were also recorded. GPS units with a frequency of 15 Hz provide a good level of reliability when measuring total distance (ICC<1.9%). However, the ICC increased in the case of peak speed measurement (ICC = 8.1%), especially when the exercise performed in speeds above 20 km/h [30]. Heart rate was monitored using a telemetric portable HR monitor system (Team Polar, Polar Electro Oy, Kempele, Finlandia).

Concentric and eccentric isokinetic peak torque at 60°/s and 180°/s and maximal isometric strength of KE and KF in both dominant and non-dominant limb was measured using an isokinetic dynamometer (Cybex 6000). Prior to testing, participants received adequate instruction and performed a familiarization protocol with submaximal repetitions as well as 10-min warm-up on a cycle ergometer (Monark 834E, Sweden) Participants, sat on the dynamometer with their back slightly reclined, their thigh fully placed on the seat (a thigh-trunk angle of 85° was adapted), were stabilized with pelvic and shoulder strapping. The rotational axis of the knee was lined up with the axis of the instrument (aligned to the lateral femoral epicondyl) with the lever arm strapped on the shank of the tested limb. Participants relaxed their limb to allow a passive measurement of the effects of gravity on the lower limb and lever arm (and a correction was applied on recorded moment of force). During isometric testing, three maximal repetitions were performed separated by 45-second intervals and the highest torque value was recorded [31]. Repetitions during which participants performed an initial countermovement were canceled and repeated. During isokinetic testing, knee's range of motion was 70° [32]. To secure knee's full extension, anatomical 90° was assessed manually with a goniometer. During the preliminary instruction and familiarization, participants were taught how to move the lever (by pushing up and pulling down) with a maximal intensity and velocity. During concentric and eccentric testing, knee extension was performed first. For eccentric testing, participants were taught how to resist the lever arm. During isokinetic testing at 60°/s and 180°/s, participants performed five maximal repetitions separated by 60-second intervals. Isometric and isokinetic testing (concentric and eccentric) in each limb were performed in a random sequence separated by a 2-minute recovery period. To ensure maximal effort, verbal encouragement was provided to all participants. The intra-class correlation coefficients for repeated measurements were: 0.92 for isometric strength, 0.91 for concentric knee flexion at 60°/s, 0.88 for concentric knee flexion at 180°/s, 0.94 for concentric knee extension at 60°/s, 0.90 for concentric knee flexion at 180°/s, 0.86 for eccentric knee flexion at 60°/s, 0.83 for eccentric knee flexion at 180°/s, 0.85 for eccentric knee extension at 60°/s, 0.88 for eccentric knee flexion at 180°/s).

### Blood sampling and assays

After an overnight fast and with participants in a semi-recumbent position, blood samples (~12 ml) were collected (7:00–8:00 a.m.) as described elsewhere [24]. A blood portion was collected into Vacutainer tubes containing ethylenediaminetetraacetic acid (EDTA) for plasma separation by centrifugation (1,370g, 4°C, 10 minutes) for the measurement of creatine kinase activity. Samples were stored in several aliquots at -75°C for later analyses. Samples thawed only once before analyzed and assays were performed in duplicate. Another blood portion (2 ml) was immediately placed in EDTA tubes to prevent clotting for the determination of complete blood count within 24h using an automated hematology analyzer (Shenzhen Mindray, BC—5500, Shenzhen, China). Blood lactate was measured with a hand-portable analyzer (Accutrend Plus, Roche Diagnostics, Basel, Switzerland) as described [33]. A Cobas Integra Plus-400 chemistry analyzer (Roche Diagnostics, Mannheim, Germany) was used to measure creatine kinase activity by an enzymatic spectrophotometric method [17]. Inter- and intra-assay coefficients of variation for all blood parameters ranged from 2.3 to 8.1% and from 2.9 to 7.5%, respectively.

### Statistical analysis

Data are presented as means±SE. In order to examine the different time point changes between the experimental and control group during the recovery period, in isokinetic performance and exercise-induced muscle damage variables, a two-way (group by time) repeated measures ANOVA with planned contrasts on different time points was used. When a significant interaction was observed, a Bonferroni correction analysis was utilized for pairwise comparisons. To determine if there was a difference between the responses of dominant limb and non-dominant limb, changes in the ratio of non-dominant limb to dominant limb values were evaluated across time.

Cluster analysis is used to group a set of objects or subjects in such a way that subjects in the same group (also named a cluster) have similar characteristics to each other compared to those in the other groups (or clusters). Cluster analysis with Ward method and squared Euklidean measure was used in order to group the subjects of EG group in two sub groups, low and high performers, based on their external load. Variables that were included in the calculation were total distance, walking, running, high intensity running, sprinting, low acceleration, moderate acceleration, high acceleration, low deceleration, moderate deceleration, high deceleration and total of acceleration and deceleration. Subsequently, a discriminant analysis was performed to identify which variables separated the groups formed by cluster analysis that revealed that players could be categorized in two groups: a) high performers and b) low performers (Table 4). The pooled within groups correlation between discriminating variables and standardized canonical discriminant functions was calculated (Table 4). The post-match period (60h) was then subdivided in two separate phases: i) performance deterioration phase: the first 12h during which performance decline reached its nadir and ii) performance recovery: 12h-60h during which performance gradually returned to baseline values. In order to examine the different time-point alterations between groups (formed by cluster analysis, i.e. high and low performers) in conventional and functional ratios at 60°/s and 180°/s during the two phases, a two-way, repeated measures ANOVA was used. Then, a stepwise multiple regression analysis (variables inserted: total distance, high-intensity running, sprinting, moderate and high acceleration/deceleration, maximal oxygen consumption and meters covered in YO-YO IR2 test) was constructed to evaluate the parameters that affected performance deterioration (period from baseline to Day 1 of recovery) and recovery (period from day 2 to day 3). Significance was accepted at p<0.05. The SPSS was used for all analyses (SPSS Inc., Chicago, IL, USA).

**Table 4:** Discrimination of players of the experimental group into a high- and low-performer subgroup based on physical activity variables indicative of match's external load as derived from cluster and discriminant analysis.

Variable	Functions^1^	Low performer subgroup	High performer subgroup
		(12 participants)	(8 participants)
**Total Distance (m)**	0.57	9,626.5 ± 611.7	11,558.5 ± 529.5
**Walking (m)**	0.46	3,106.2 ± 194.7	3,688.4 ± 248.3
**Jogging (m)**	0.57	3,937.2 ± 250.2	4,727.4 ± 216.6
**Running (m)**	0.57	1,771.3 ± 112.5	2126.8 ± 97.4
**High Intensity Running (m)**	0.56	572.7 ± 36.1	688.2 ± 33.5
**Sprinting (m)**	0.16	239.1 ± 27.5	265.2 ± 29.6
**High Speed Running (m)**	0.40	811.8 ± 60.1	953.4 ± 60.6
**Low acceleration (m)**	-0.30	682.4 ± 97.5	538.9 ± 41.7
**Moderate acceleration (m)**	0.33	176.3 ± 30.1	245.6 ± 43.3
**High acceleration (m)**	0.34	125.5 ± 43.1	201.7 ± 27.7
**Low deceleration (m)**	-0.21	580.1 ± 71.5	497.5 ± 61.3
**Moderate deceleration (m)**	0.21	164.6 ± 35.6	205.5 ± 29.3
**High deceleration (m)**	0.44	113.9 ± 23.3	176.6 ± 26.1
**Sum of high acceleration and deceleration (m)**	0.39	239.4 ± 64.6	378.2 ± 52.2

^1^ Within-groups correlations between discriminating variables and standardized canonical discriminant functions.

## Results

Groups demonstrated comparable age, training age, body weight and BMI, body fat percentage, VO_2max_ and YO-YO IR2, resting and maximal heart rate (Table 1).

### Match evaluation

The match increased blood lactate (P = .001) as well as mean (P = .001) and maximal (P = .001) heart rate (Table 5). Table 6 presents GPS-measured activity profile during the match. Activity profile during training was comparable among groups (Table 3).

**Table 5:** The physiological profile of the football match.

	*Rest*	Game Responses
**Mean heart rate (beat/min)**		
C	**68.2 ± 6.4**	N/A
EG	**66.1 ± 4.2**	164.9 ± 8.2^1^ ^,^ ^2^
**Peak heart rate (beat/min)**		
C	**N/A**	N/A
EG	**N/A**	193.8 ± 6.5^1^ ^,^ ^2^
**Lactate (mM)**		
C	**1.23 ± 0.2**	N/A
EG	**1.14 ± 0.2**	5.02 ± 1.0^1^ ^,^ ^2^

^1^Significant difference with baseline

^2^significant difference between groups

N/A, not applicable.

**Table 6:** GPS-measured activity profile of the experimental group during the football match.

Activity variables	Distance covered (m)
**Total distance (m)**	10,399.3 ± 1,123.8
**Distance covered at 0–7.2 km/h (m)**	3,339.1 ± 361.0
**Distance covered at 7.3–14.4 km/h (m)**	4,253.3 ± 459.6
**Distance covered at 14.5–19.8 km/h (m)**	1,913.5 ± 206.8
**Distance covered at 19.9–25.2 km/h (m)**	618.9 ± 67.4
**Distance covered at >25.2 km/h (m)**	249.5 ± 30.5
**Distance covered at accelerations of 1–2 m/s^2^**	625.0 ± 106.6
**Distance covered at accelerations of 2–3 m/s^2^**	204.0 ± 49.3
**Distance covered at accelerations of >3 m/s^2^**	155.9 ± 53.1
**Distance covered at decelerations of 1–2 m/s^2^**	547.0 ± 77.9
**Distance covered at decelerations of 2–3 m/s^2^**	180.9 ± 38.4
**Distance covered at decelerations of >3 m/s^2^**	138.9 ± 39.5

GPS, global positioning system.

### Temporal responses of muscle damage markers

**Fig 2:**
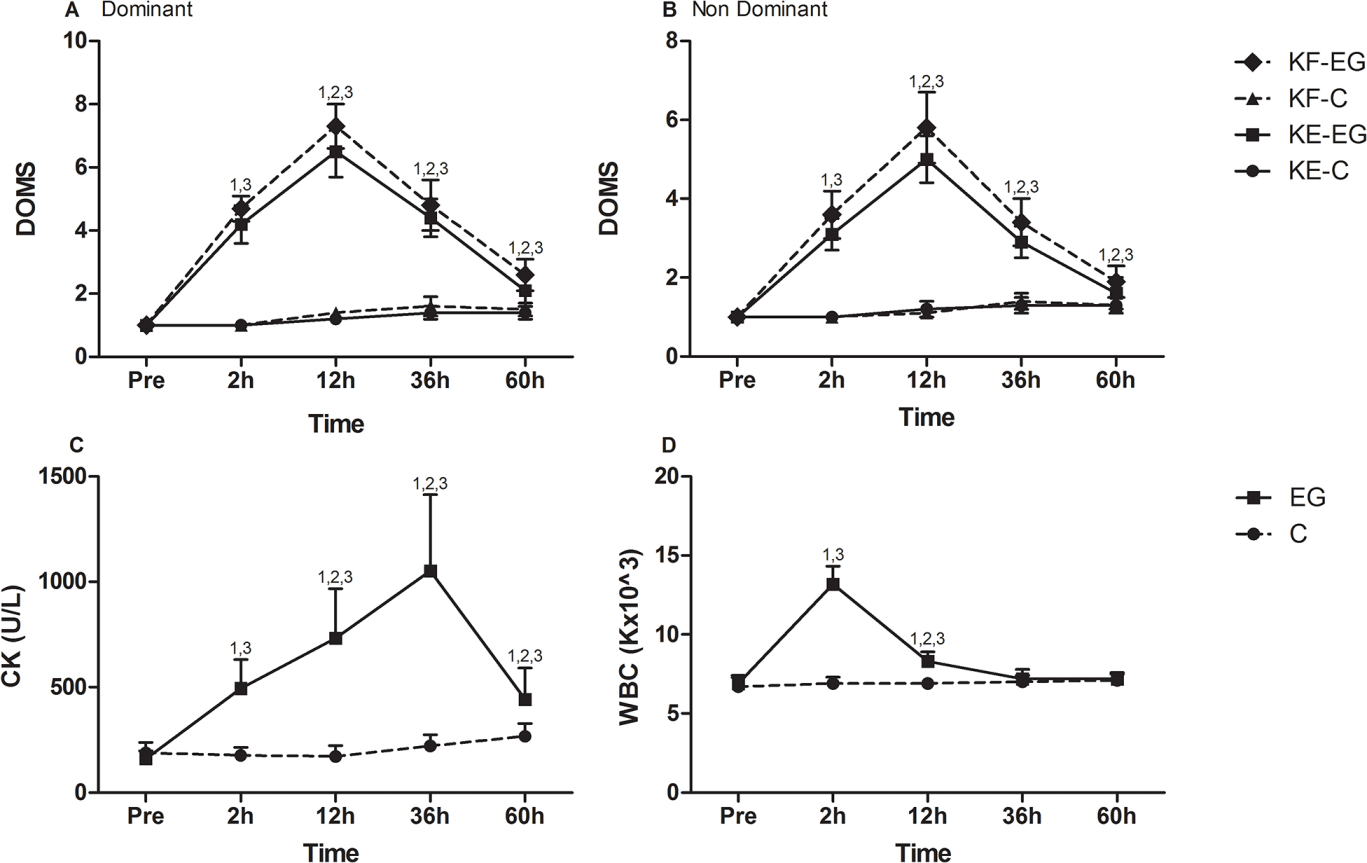
Changes of soreness (A-B), CK activity (C) and leukocyte counts (D) following a football match. MS, muscle soreness; CK, creatine kinase activity; h, hours; C, control group; EG, experimental group; KE-C, knee extensors of control group; KE-EG, knee extensors of experimental group; KF-C, knee flexors of control group; KF-EG, knee flexors of experimental group; ^1^Significant difference with baseline; ^2^significant difference between groups; ^3^significant difference between dominant and non-dominant limb at corresponding time; ^4^greater decline in functional ration compared to conventional ratio at corresponding time;^5^greater decline at 180°/s compared to that at 60°/s at corresponding time.

Fig 2 illustrates changes in muscle damage markers and leukocyte counts. In C, soreness of both KF (dominant limb: P = 0.041; non-dominant limb: P = .016) and KE (dominant limb: P = 0.002; non-dominant limb, P = .018) was elevated in both limbs only at 60h. Soreness of KF and KE in EG increased in both limbs throughout recovery (KF: P = .001 both limbs throughout recovery; KE: P = .001 both limbs at 12h and 36h and for dominant limb at 60h; P = .01 for the non-dominant limb at 60h). The ratio of KE to KF soreness values declined at 2h and 60h for dominant limb (2h, P = .001; 60h, P = .014) and at 2h, 36h and 60h for non-dominant limb (P = .001) suggesting a more pronounced elevation of MS for KF than KE at these time points. Creatine kinase activity increased throughout recovery only in EG (P = .001 throughout recovery). Leukocyte counts increased only in EG at 2h (P = .001) and 12h (P = .001) of recovery and normalized thereafter. Soreness (throughout recovery), creatine kinase activity (throughout recovery) and leukocyte count (post-match and at 12h) values in EG were always higher than those in C (P < .05).

### Temporal responses of strength performance

**Fig 3:**
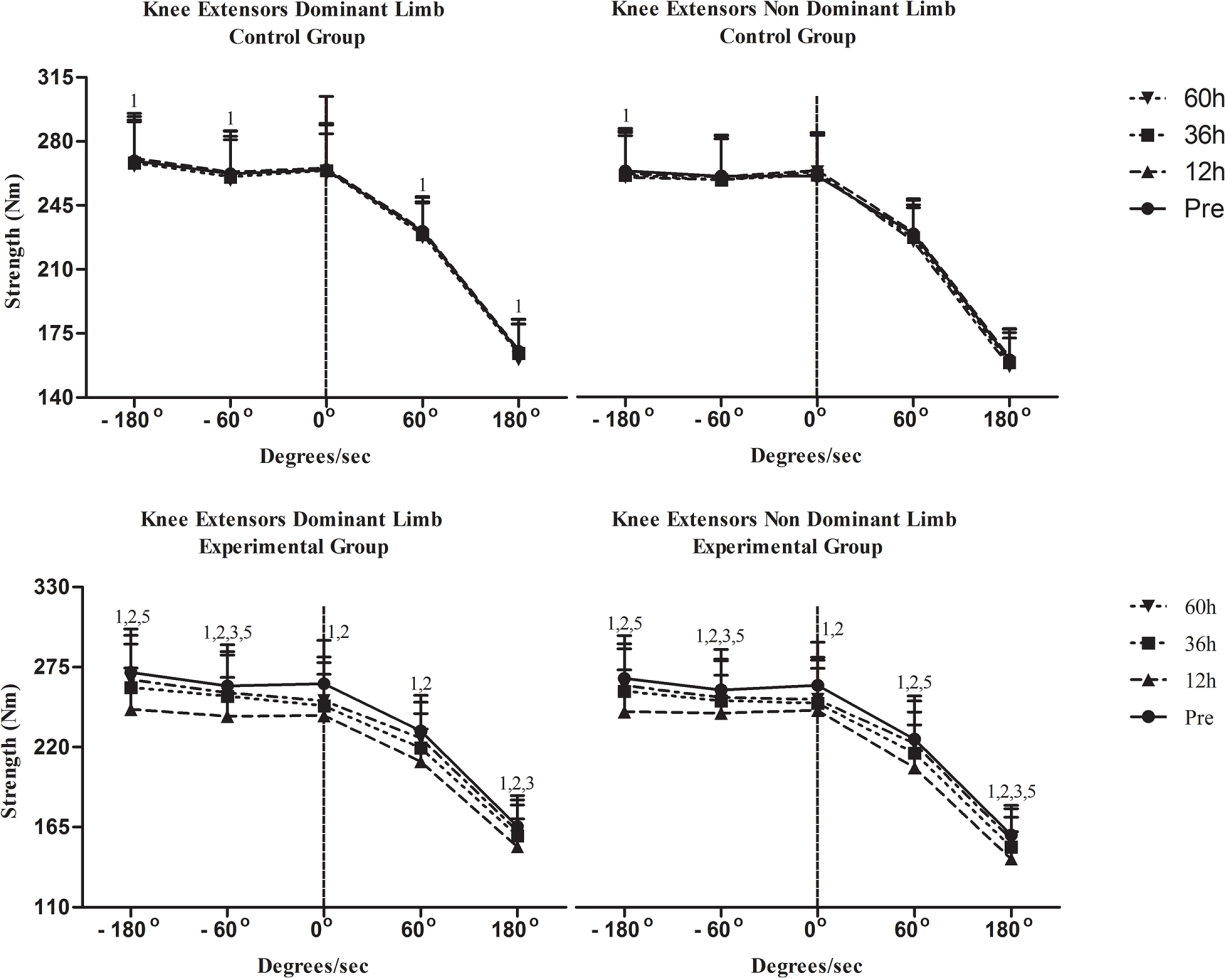
Changes of strength performance of knee extensors in response to a football match. h, hours; ^1^Significant difference with baseline; ^2^significant difference between groups; ^3^significant difference between dominant and non-dominant limb at corresponding time; ^4^greater decline in functional ration compared to conventional ratio at corresponding time;^5^greater decline at 180°/s compared to that at 60°/s at corresponding time.

**Fig 4:**
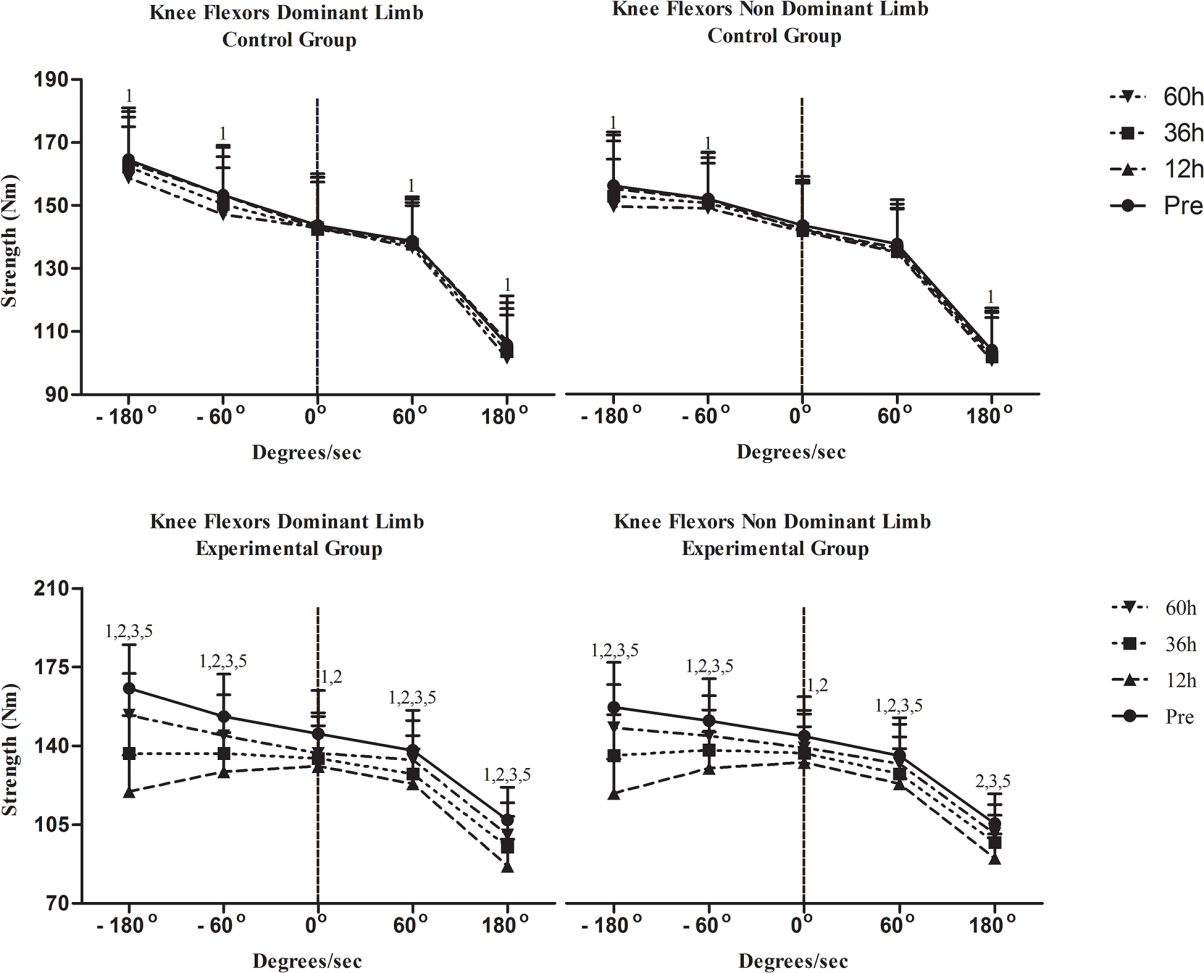
Changes of strength performance of knee flexors in response to a football match. h, hours; ^1^Significant difference with baseline; ^2^significant difference between groups; ^3^significant difference between dominant and non-dominant limb at corresponding time; ^4^greater decline in functional ration compared to conventional ratio at corresponding time;^5^greater decline at 180°/s compared to that at 60°/s at corresponding time.

Changes in isometric strength are shown in Fig 3 for KE and in Fig 4 for KF. Isometric performance remained unchanged in C. Isometric strength of KE and KF declined similarly in both limbs at 12h (KE: dominant limb, P = .017; non-dominant limb, P = .001; KF: dominant limb, P = .002; non-dominant limb, P = .005), 36h (P = .001 for KE and KF of both dominant limb and non-dominant limb) and 60h (P = .001 for KE and KF of both dominant limb and non-dominant limb). Isometric strength values in EG were always higher (throughout recovery except for KF in dominant limb at 60h and non-dominant limb at 36h and 60h) than those in C (P < .05).

**Fig 5:**
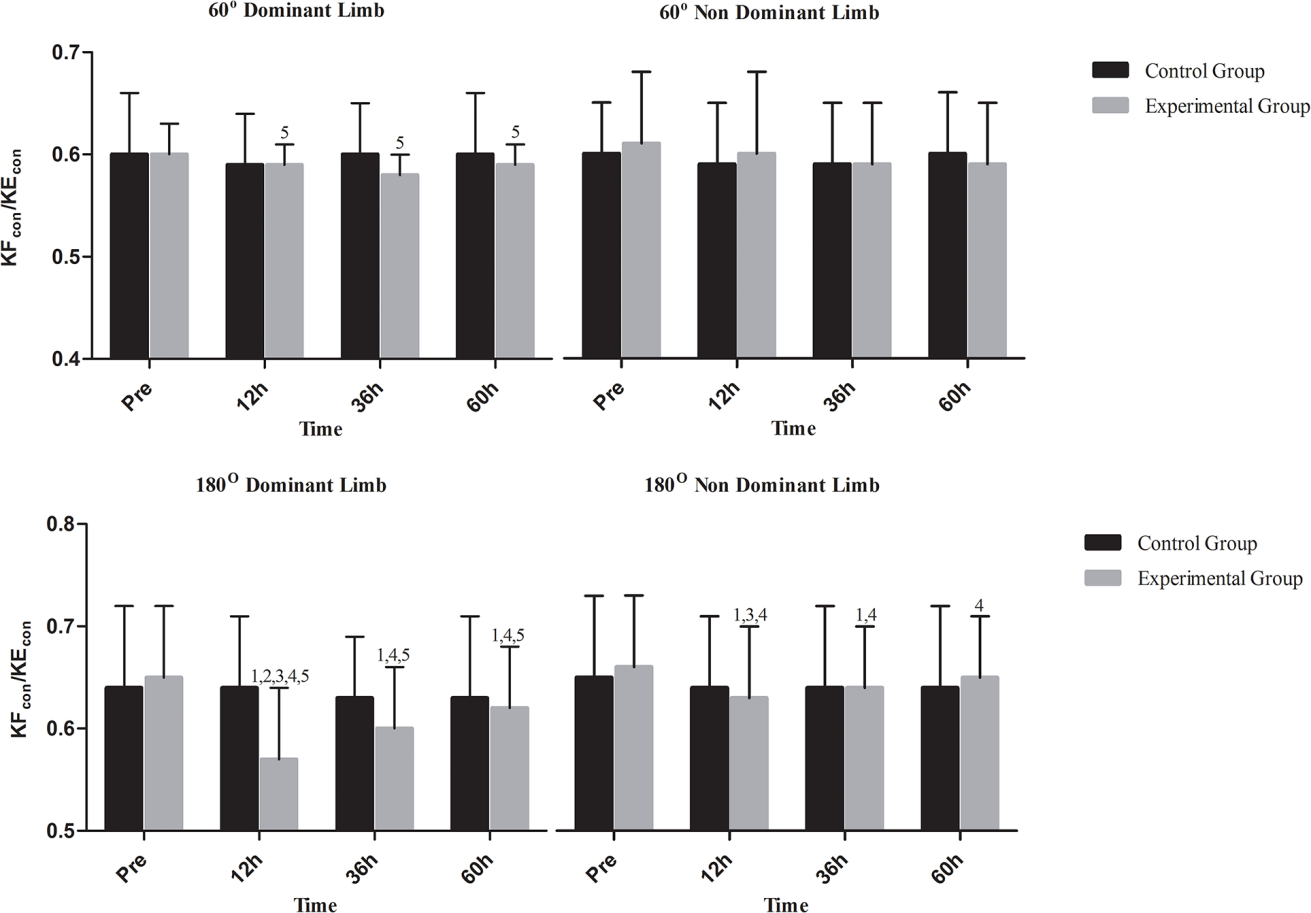
Changes of conventional ratio in response to a football match. KF_con_/KE_con_, conventional ratio; h, hours; ^1^Significant difference with baseline; ^2^significant difference between groups; ^3^significant difference between dominant and non-dominant limb at corresponding time; ^4^greater decline in functional ration compared to conventional ratio at corresponding time;^5^greater decline at 180°/s compared to that at 60°/s at corresponding time.

**Fig 6:**
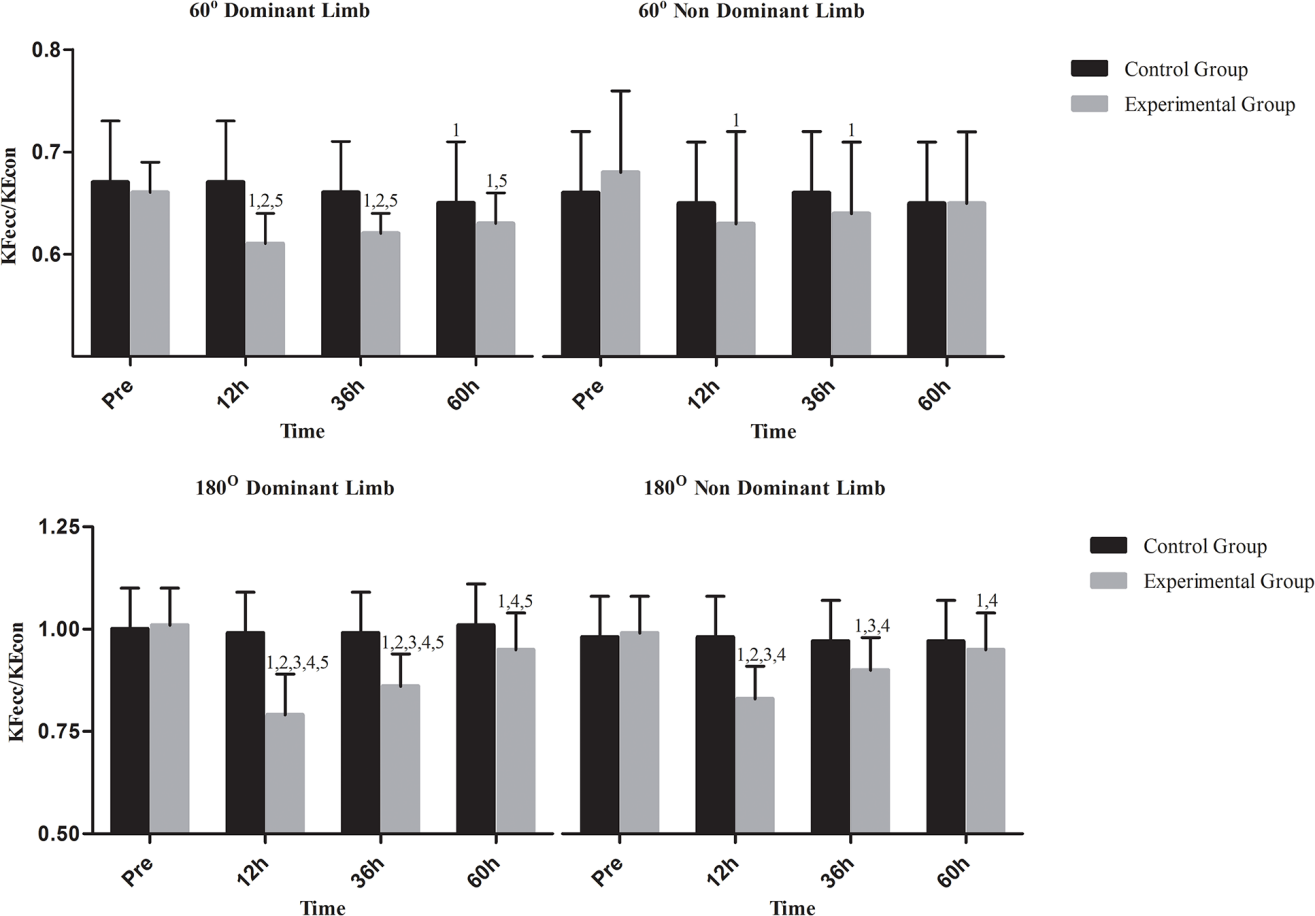
Changes of functional ratio in response to a football match. KF_ecc_/KE_con_, functional ratio; h, hours; ^1^Significant difference with baseline; ^2^significant difference between groups; ^3^significant difference between dominant and non-dominant limb at corresponding time; ^4^greater decline in functional ration compared to conventional ratio at corresponding time;^5^greater decline at 180°/s compared to that at 60°/s at corresponding time.

Changes in isokinetic peak torque at 60°/s are shown in Fig 3 for KE and Fig 4 for KF. In C, concentric peak torque of both KE and KF declined only in dominant limb at 60h (KE: P = .001; KF: P = .02) whereas eccentric peak torque declined in KE of dominant limb (P = .001) and KF of both limbs at 60h (P = .001 for both limbs). Concentric peak torque of KE and KF in EG decreased in both limbs at 12h (P = .001 for KE and KF in both limbs), at 36h (KE: dominant limb, P = .009; non-dominant limb, P = .021; KF: dominant limb, P = .002; non-dominant limb, P = .013) and 60h (P = .001 for KE and KF in both limbs). When groups were compared, EG demonstrated lower concentric peak torque for KE and KF in both limbs throughout recovery except from those at 60h (P < .05). Eccentric peak torque of KE and KF decreased in both limbs at 12h, 36h and 60h (P = .001 for KE and KF in both limbs at all time points of measurement during recovery). Eccentric peak torque of KF decreased similarly in both limbs at 12h, 36h and 60h (P = .001 for both limbs throughout recovery). When groups were compared, EG exhibited a greater decline of eccentric peak torque of KE and KF in at 12h and 36h in both limbs (P < .05). KF_con_/KE_con_ (Fig 5) remained unaffected in both limbs in both groups throughout recovery. In C, KF_ecc_/KE_con_ (Fig 6) declined only in dominant limb at 60h (P = .001). In EG, KF_ecc_/KE_con_ (Fig 6) declined in dominant limb at 12h, at 36h and 60h (P = .001 throughout recovery) and in non-dominant limb at 12h and 36h (P = .001 for both time points). The decline of KF_ecc_/KE_con_ was more pronounced in EG than C at 12h (P = .005) and 36h (P = .026) in dominant limb.

Changes in relative isokinetic peak torque at 180°/s are shown in Fig 3 for KE and Fig 4 for KF. In C, there was a decline of concentric peak torque of KE (dominant limb, P = .008; non-dominant limb, P = .001) and KF at 60h (P = .001 for both limbs) as well as of eccentric peak torque of KF and KE at 60h (P = .001 for KE and KF of both limbs). In EG, concentric peak torque of KE decreased in both limbs at 12h (P = .001 for both limbs), 36h (P = .001 for both limbs) and 60h (dominant limb: P = .047; non-dominant limb: P = .001). Concentric peak torque of KF decreased in both limbs also throughout recovery (P = .001 for both limbs throughout recovery). When groups were compared, EG demonstrated a greater decline of concentric peak torque of KE and KF than C in both limbs at 12h and 36h (P < .05). Eccentric peak torque of KE decreased in both limbs at 12h, 36h and 60h (P = .001for both limbs throughout recovery). Eccentric peak torque of KF decreased in both limbs throughout recovery (P = .001 for both limbs throughout recovery). The EG demonstrated a greater decline of eccentric peak torque of KE and KF in both limbs at 12h and 36h (P < .05). Both conventional and functional ratios (Figs 5 and 6) remained unaltered in C. In EG, a marked decline was observed for both KF_con_/KE_con_ (Fig 4, 12h: P = .001for both limbs; 36h: dominant limb, P = .001; non-dominant limb: P = .027; 60h: dominant limb, P = .001) and KF_ecc_/KE_con_ throughout recovery (Fig 5; P = .001for both limbs throughout recovery). The EG demonstrated a more pronounced decline of KF_con_/KE_con_ than C at 12h in dominant limb (P < .05). The decline of KF_ecc_/KE_con_ was greater in EG in dominant limb at 12h and 36h (P < .05) and at 12h in non-dominant limb (P < .05).

### Comparison of dominant and non-dominant limb responses

In respect to MS development, the ratio of non-dominant limb to dominant limb soreness values demonstrated a greater decline at 2h (P = .027), 12h (P = .001) and 36h (P = .008) for KF and at 2h (P = .001), 12h (P = .001), 36h (P = .003) and 60h (P = .001) for KE suggesting that dominant limb was more affected than non-dominant limb. At 60°/s, dominant limb demonstrated a greater decline of concentric peak torque of KF at 12h (P = .013), at 36h (P = .006), eccentric peak torque of KE at 12h (P = .028) and eccentric peak torque of KF at 12h (P = .001), 36h (P = .001) and 60h (P = .036) than non-dominant limb. At 180°/s, dominant limb demonstrated a greater decline of concentric peak torque of KE and KF throughout recovery (KE: 12h, P = .001; 36h, P = .002; 60h, P = .04; KF: 12h, P = .001; 36h, P = .001; 60h, P = .044), eccentric peak torque of KF throughout recovery (12h: P = .001; 36h: P = .002; 60h: P = .019), KF_con_/KE_con_ at 12h (P = .001) and KF_ecc_/KE_con_ at 12h (P = .001) and 36h (P = .002) than non-dominant limb.

### Comparison of conventional and functional ratio responses

To determine if there was a difference between the responses of conventional and functional ratio, changes in the ratio of KF_ecc_/KE_con_ to KF_con_/KE_con_ values were evaluated across time. This ratio at 180°/s demonstrated a marked reduction throughout recovery (12h: dominant limb, P = .001; non-dominant limb, P = .001; 36h: dominant limb, P = .001; non-dominant limb, P = .001; 60h: dominant limb, P = .001; non-dominant limb, P = .033) indicating that KF_ecc_/KE_con_ is more affected than KF_con_/KE_con_ in response to a football match.

### Comparison of strength responses at 60°/s and 180°/s

To determine if there was a difference between the responses at 60°/s and 180°/s, changes in the ratio of 60°/s and 180°/s values in EG were evaluated across time. It was revealed that decrements in isokinetic performance in response to a football match were greater at 180°/s for concentric peak torque of KE in non-dominant limb at 12h (P = .016), concentric peak torque of KF of both limbs at all times (dominant limb: 12h, P = .001; 36h, P = .001; 60h, P = .001—non-dominant limb: 12h, P = .001; 36h, P = .001; 60h, P = .034), eccentric peak torque of KE at 12h (dominant limb, P = .01; non-dominant limb, P = .001) and 36h (dominant limb, P = .006), eccentric peak torque of KF at 12h (dominant limb, P = .001; non-dominant limb, P = .001;), 36h (dominant limb, P = .001; non-dominant limb, P = .001) and 60h (dominant limb, P = .014), KF_con_/KE_con_ in dominant limb only (12h, P = .001; 36h, P = .001; 60h, P = .003) and KF_ecc_/KE_con_ in dominant limb only (12h, P = .001; 36h, P = .001; 60h, P = .034).

### Factors associated with recovery kinetics of strength performance in response to a football match

Results showed that those who were more active during the match (high performers) compared to those who were less active (low performers, Table 4) had similar ratios at 60°/s but differed in both ratios (conventional ratio: P = .031; functional ratio: P = .002) at 180°/s during both phases suggesting that match physical activity may affect recovery of isokinetic performance. Stepwise regression analysis (Table 7) indicated that: a) during the first phase (0–12h) only the decline of functional ratio could be predicted at 60°/s by sprinting distance whereas at 180°/s the decline of conventional and functional ratio could be predicted by high acceleration distance and b) during the second phase (12h-60h), the recovery of both ratios could be predicted by YO-YO IR2 scores (both for 60°/s and 180°/s).

**Table 7:** Stepwise linear regression model summary for each component predictors.

Variable	Predictor	R^2^	Equation
**60** ^**o**^ **/sec conventional ratio**			
Deterioration phase (Pre-12h)	-	-	-
Recovery phase (12–60h)	Yo-Yo IR2 scores	0.29 (F = 8.59, P<.05)	F_(x)_ = 10.98–0.009(x)
**60** ^**o**^ **/sec, Functional ratio**			
Deterioration phase (Pre-12h)	Sprinting distance	0.47 (F = 17.56, p<.05)	F_(x)_ = -3.1+0.43(x)
Recovery phase (12–60h)	Yo-Yo IR2 scores	0.54 (F = 23.21, p<.05)	F_(x)_ = 15.93–0.016(x)
**180** ^**o**^ **/sec, Conventional ratio**			
Deterioration phase (Pre-12h)	High acceleration distance	0.67 (F = 36.29, p<.05)	F_(x)_ = 4.4+0.05(x)
Recovery phase (12–60h)	Yo-Yo IR2 scores	0.51 (F = 21.08, p<.05)	F_(x)_ = -28.89–0.028(x)
**180** ^**o**^ **/sec, Functional ratio**			
Deterioration phase (Pre-12h)	High acceleration distance	0.72 (F = 45.2, p<.05)	F_(x)_ = 9.25+0.076(x)
Recovery phase (12–60h)	High acceleration	0.71 (F = 42.94, p<.05)	F_(x)_ = -1.19–0.12(x)

## Discussion

Football is one of the most popular, if not the most, sports in the world with millions of people participating in formal and casual competition. Thigh injuries and especially hamstring strains are the most prevalent ones in football resulting in significant loss of practice and match hours. However, limited data exist regarding temporal changes of KF and KE isokinetic strength performance after a match. Previous investigations used mostly intermittent running protocols on the field or in the laboratory that consist of running at pre-determined maneuvers and intensities but lack random, non-linear runs characterized by rapid, eccentric in nature, acceleration/deceleration motions. These protocols also lack other psychological and environmental factors that affect metabolic and biomechanical variables that predispose athletes to injuries [9]. Data on how a football match affects different types of strength (isometric vs. concentric vs. eccentric) at various knee movement velocities (low vs. high) and in respect to limb dominance are lacking [6–7, 16, 18, 19, 34–36]. Furthermore, limited information exists regarding the independent effects of football practice on strength performance responses. We found that a football match induced a prolonged decline of strength performance of muscles involved in knee motions in a limb- and velocity-specific manner and that recovery kinetics of strength performance may be related to match activity profile and players' fitness status.

### Match activity

Although semi-professional players participated, the experimental match resembled elite football competition since athletes displayed values of match-related physical activity and physiological response (lactate, heart rate) that approximated those reported for elite match-play [1, 37]. Furthermore, the match provoked an increase in muscle damage and inflammatory markers similar to that previously reported in response to official football matches [18, 34]. Collectively, these results suggest that the experimental match reflected a competitive football scenario.

### Inflammatory and muscle damage markers

Muscle damage markers (soreness and creatine kinase activity) demonstrated a typical response usually seen after a football match [7, 19, 34] that peaked within the first 24h after the match and declined thereafter. Creatine kinase activity increased ~2-5-fold after the game compared to baseline at 36h. This is considered a physiological post-game response for creatine kinase activity since its reference intervals for football players are exceptionally high probably due to the nature of football training and competition [38]. The inflammatory response, as evidenced by the leukocyte count, was short-lived (12h) and in agreement with previous findings [7, 19, 34]. Interestingly, individuals reported more soreness of KF than KE at all times which is in accordance with the greatest decline of KF eccentric and concentric strength during the post-match period. Although hamstrings' soreness and performance decline has been reported following football-like intermittent-type running and after sport events characterized by strong eccentric component such as basketball and team handball [24, 39], such findings were not seen after a football match [35]. This is not surprising since both the magnitude and the time-course of soreness is poorly correlated with fluctuations of muscle function following muscle damaging exercise [40]. In fact, soreness peaks after the onset of histologically-assessed muscle damage and strength decline [40]. KF may be more prone to exercise-induced damage due to their intense eccentric involvement during landing in actions such as sprinting, accelerations/decelerations and cutting maneuvers in an attempt to prevent knee's overextension and hip's flexion [9, 11, 12]. Strength decline, which is considered as a valid marker of muscle damage [40], demonstrated a marked decline for both knee flexors and extensors during recovery with flexors demonstrating a greater loss than extensors indicating the occurrence of muscle damage for both muscle groups.

### Temporal changes of strength performance

In this study, strength performance was evaluated by isokinetic testing since it has been shown to be an effective diagnostic mode of KF and KE strength imbalances that predispose football players to knee injuries [41]. In fact, normalization of such imbalances or asymmetries between KE and KF led to prevention of subsequent knee re-injuries [41]. All types of strength (isometric, concentric, eccentric) of both KE and KF reached their lowest value 12h after the match and recovered thereafter without, however, returning at baseline 60h after the match. Most previous studies agree that strength reaches its nadir 12–24h after a match [6, 34–36]. However, most studies suggest that strength recovers 36h to 48h after a match [6, 34–36]. In accordance with our results, a recent study [16] reported more prolonged strength deterioration (72h). Prolonged strength decline may be explained by a decrease in muscle voluntary activation due to soreness-related inhibition [36]. A plausible explanation for discrepancies in the time-frame of strength decline after a football match may be related to the presence or absence of daily practice during the post-match recovery period and the nature of the experimental game (i.e. friendly or competitive). In this study, athletes trained daily after the match and training might have extended the period of strength reduction. The fact that the C demonstrated a performance decline of concentric and eccentric peak torque, especially at high velocity testing, at 60h (after the second and more intense practice), provides further support to this claim, i.e. intense football practice may independently predispose to strength decline of knee extensors and flexors that contract both concentrically and eccentrically during intense cyclic running football activity. Future research should investigate strength performance fluctuations due to practice load during a training microcycle.

### Comparison of strength responses at 60°/s and 180°/s

Our data suggest that strength deterioration was velocity-specific since KF_ecc_/KE_con,_ KF_con_/KE_con_, eccentric and concentric peak torque of both KF and KE were more pronounced at 180°/s as compared to 60°/s. Greig et al. [42] reported that KF_ecc_/KE_con_ is more impaired at fast compared to slow velocities. The decline observed in that study was similar to that seen here (12% at 60°/s, 23% at 180°/s). This data reveals a specificity effect since football consists of a large number of explosive ballistic motions during which knee velocity reaches values of 745–860°/s and 860–1720°/s during knee flexion and extension, respectively [43].

### Conventional vs. functional ratio responses

Concentric peak torque of KF declined at a greater extent than concentric peak torque of KE at 60°/s (3.1%-10.6% for KF vs. 2.1%-9.1% for KE) and 180°/s (5.6%-16.1% for KF vs. 1.8%-8% for KE). Likewise, eccentric strength of KF demonstrated a far more pronounced reduction than eccentric peak torque of KE at 60°/s (6.3%-19% for KF vs. 2%-8.3% for KE) and 180°/s (7.2%-27.7% for KF vs. 1.9%-9.4% for KE). Similar results have been reported following football-like intermittent running [13] suggesting that KF demonstrate greater strength deficits than KE (also supported by higher soreness values for KF), especially at high contraction velocities, for prolonged periods after or even during a football match [15]. These results also suggest that eccentric strength of KF is more susceptible to deterioration than concentric strength in response to football activity. Compared to concentric contraction, eccentric muscle elongation elicits a higher tension per cross-sectional area of active musculature thereby resulting in greater ultrastructural damage of myofibre [44]. Due to the protective role of KF towards prevention of knee hyperextension, these deficits may increase knee's susceptibility to injury, especially in response to fatigue induced by condensed training and competition during in-season. A greater decline of KF strength compared to KE may compromise knee's control and stability and thus increase injury risk during intense training usually scheduled 30–80h post-match within an in-season microcycle. Therefore, training and recovery strategies (such as water therapy and cryotherapy) should emphasize the restoration of eccentric strength of KF in order to maintain an optimal KF/KE ratio.

The fact that KF_con_/KE_con_ remained unaltered at 60°/s and demonstrated a decline of smaller magnitude than KF_ecc_/KE_con_ at 180°/s also supports the notion that eccentric peak torque of KF is more affected by intense football activity suggesting that the functional ratio may be a better alternative for monitoring football-related fatigue than the conventional ratio. Although there is no previous information on how match activity affects these two ratios, results from studies that utilized intermittent running tests to simulate football activity are in agreement with our findings [14, 45]. In fact, football-like activity has been to shown to leave concentric peak torque of KF unaffected [42, 46]. These results may also be attributed to hamstrings' greater proportion of type II myofibre that are characterized by a greater fatigability than type I fibers [13]. Hamstrings contract eccentrically during actions such as sprinting, kicking, tackling, changing directions etc. to counterbalance anterior shear forces produced by KE and decelerate thigh and leg movements prior to foot contact [8] rendering them susceptible to tear thereby reducing their input to stabilize the knee and thus placing more stress on the ACL during states of fatigue. The size of reduction of KF_ecc_/KE_con_ in this study was smaller than that observed after an intermittent shuttle test [14] but similar to that recorded after treadmill running [13]. Differences in protocols used and musculoskeletal fitness status of participants may explain such discrepancies. All together, these findings suggest that football players may need to eccentrically train their KF at high velocities such as those obtained during the late-stance phase of sprinting [43]. Additional eccentric training of KF during the pre-season has been shown to decrease in-season knee injuries [47].

### Dominant vs. non-dominant limb responses

Football players have been suspected for bilateral asymmetries due to a greater unilateral stress of dominant leg's hamstrings when acting to stabilize the knee when players perform football-specific actions such as passing and kicking tackling [48]. However, participants in this study demonstrated a ≤10% strength (isometric and isokinetic) asymmetry between dominant limb and non-dominant limb at low- and high-velocity testing at baseline (prerequisite for participation) and thus any differences observed during the post-match period could be attributed mainly to match or practice load.

Dominant limb demonstrated not only higher soreness values for both KE and KF but also a greater decline of strength at 0°/s (both KE and KF), 60°/s (concentric peak torque of KE, eccentric peak torque of KF and KF_ecc_/KE_con_) and 180°/s (concentric peak torque of KE and KF, eccentric peak torque of KF, KF_ecc_/KE_con,_ KF_con_/KE_con_). Although they had only one post measurement, two previous studies that used protocols simulating football reported similar findings [14, 46] while a third one failed to detect statistically meaningful differences between limbs despite a 6% greater decline in dominant limb [15]. In fact, male football players tend to injure their dominant limb more [9]. Our results indicate that dominant limb is affected more than non-dominant limb, especially KF strength at high velocity, for as long as 60h post-match. These findings may be explained by the fact that players tend to use their dominant limb more as their pivot limb during technical actions, agility running (especially towards the end of a run to decelerate the body), tackling, dribbling, passing and kicking [14]. When these actions include ball handling they require more energy and force production [14] and thus placing asymmetric demands on dominant limb. This notion must be taken into consideration when investigating potential match recovery strategies for affected muscles (such as water therapy, cryotherapy, etc.).

### Factors related to recovery kinetics of strength performance

Cluster and regression analyses revealed that those who covered the largest distances sprinting and accelerating also demonstrated the greatest decline of functional ratio at 60°/s and of both ratios at 180°/s during the first 12h after the match. Similar results have been obtained during the first 24h after a football match by a very recent and interesting study that used video analysis [16]. These results suggest that the magnitude of muscle performance deterioration during the first 24h after a match may be attributed to the distances covered by sprinting and maximal accelerations during the match. In agreement with our results, performance deterioration after a competitive event was highly correlated with collision, tackle and acceleration number during rugby or Australian football match [49, 50]. These activities are all characterized by intense eccentric actions that induce considerable muscle damage and soreness. Interestingly, these two studies in Australian football and rugby [49, 50] observed no other relationships between playing actions and recovery kinetics beyond 24h of recovery. In this study we separated recovery into two distinct phases; the first 12h during which maximal performance decline is observed following a downward slope and a later two-day phase during which performance recovers following an upward slope. Slopes of these two phases may be related to different factors. Our analyses revealed that players with a higher Yo-Yo IR2 testing performance tend to recover faster 12h-60h after the match suggesting that football-specific conditioning may be crucial in this aspect. Yo-Yo IR2 can distinguish players of various performance levels and is highly correlated with distance covered with high-speed running during a match [51]. The protective role of training is also illustrated by findings suggesting that eccentric training not only limits muscle damage but also enhances its recovery rate due to increases of satellite cell content and activation [52]. Furthermore, an inverse relationship was observed between maximal runs/accelerations and recovery of functional performance at 180°/s during the 12h-60h period suggesting that match activity may not only affect subsequent strength deterioration but also limit its recovery. Furthermore, the degree of familiarization with football activity related eccentric activity, expressed as number of soccer matches performed by players during a season, may provide protection to skeletal muscle from strength attenuation and enhance its recovery following a football game [53]. Football players with a smaller number of games per season may be more susceptible to knee flexor and extensor strength losses after a game and a slower recovery rate, a concept that needs further investigation.

### Perspectives

Injuries of thigh musculature are the most prevalent during football match-play and training and may lead to ACL rupture during powerful ballistic motions with strong eccentric component. This study revealed that the decline of strength performance of flexors and extensors KE peaks 24h after a football match and its magnitude is greater for the flexors than extensors. Strength deterioration may be more pronounced at higher knee velocities and for dominant limb as compared to slower knee velocities and non-dominant limb. Our results also suggest that rate of strength performance deterioration may be affected by the amount of sprints and accelerations a player performs during match-play while the rate of strength recovery is positively related to players' level of football-specific conditioning. Data indicate that eccentric training of hamstrings could be used as a strategy to prevent large deterioration of eccentric peak torque of knee flexors following competition and training. This investigation also showed that the functional ratio may be more beneficial than the conventional ratio for practitioners to monitor strength deficits due to football-related fatigue for injury prevention during in-season. Although high R squared and F values were obtained in our regression analysis and a large number of comparisons have been made, a limited number of subjects were used for the number of predictor variables used in our model [54]. Further studies that will recruit a cohort of a larger sample size are needed in order to confirm the results presented by this study. Another potential limitation of this study is that participants were not professional or elite-level players who may demonstrate different recovery potential and undergo a greater football stress during a season. Another limitation is that participants in this study were healthy and with no asymmetries whereas those with musculoskeletal problems are more susceptible to match and practice injuries. Future research needs to address the post-match recovery kinetics of flexors and extensors strength of elite-level players with muscular imbalances and bilateral limb asymmetries.

## Supporting Information

S1 File Includes data of participants' characteristics as well as practice and game physical activity data. (PDF)

S2 File Includes data on muscle damage and strength performance responses. (PDF)
