# Correction: Recovery Kinetics of Knee Flexor and Extensor Strength after a Football Match

**DOI:** 10.1371/journal.pone.0133459

**Published:** 2015-07-15

**Authors:** Dimitrios Draganidis, Athanasios Chatzinikolaou, Alexandra Avloniti, José C. Barbero-Álvarez, Magni Mohr, Paraskevi Malliou, Vassilios Gourgoulis, Chariklia K. Deli, Ioannis I. Douroudos, Konstantinos Margonis, Asimenia Gioftsidou, Andreas D. Flouris, Athanasios Z. Jamurtas, Yiannis Koutedakis, Ioannis G. Fatouros

The 12^th^ author’s name is spelled incorrectly. The correct name is: Andreas D. Flouris.
