# Supplementary material for: Recovery Kinetics of Knee Flexor and Extensor Strength after a Football Match
Source: PLoS One. 2015 Jun 4;10(6):e0128072. doi: 10.1371/journal.pone.0128072 (PMC4456353; doi:10.1371/journal.pone.0128072)
Supplement: S1 File — (PDF) [file pone.0128072.s001.pdf]

SUBJECT ID	AGE (yrs)	TRAINING AGE (yrs)	WEIGHT (kg)	HEIGHT (m)	BMI (kg/m^2)	BODY FAT (%)
1	22,1	11,8	72,1	1,79	22,5	5,6
2	28,3	10,1	77,6	1,83	23,2	6,8
3	19,9	10,5	71,8	1,76	23,2	6,4
4	18,9	9,9	76,2	1,82	23,0	5,7
5	19,9	10,5	74,2	1,79	23,2	10,6
6	19,9	9,3	74,4	1,85	21,7	5,4
7	22,0	9,2	79,5	1,88	22,5	9,4
8	22,1	10,1	89,1	1,93	23,9	5,4
9	25,2	11,1	77,3	1,82	23,3	5,7
10	22,0	10,3	67,7	1,72	22,9	6,8
11	27,3	11,1	79,6	1,84	23,5	7,7
12	23,1	11,4	74,5	1,80	23,0	6,3
13	23,1	10,7	81,5	1,89	22,8	6,5
14	23,1	8,7	73,9	1,79	23,1	9,7
15	23,1	11,6	69,7	1,75	22,8	7,5
16	28,4	12,4	68,7	1,74	22,7	7,0
17	23,1	10,0	81,9	1,87	23,4	10,5
18	22,0	9,7	77,5	1,83	23,1	9,1
19	23,1	9,9	61,2	1,68	21,7	6,4
20	25,2	10,4	80,1	1,86	23,2	7,2
21	22,5	11,5	73,1	1,78	23,1	6,0
22	23,5	12,5	76,2	1,81	23,3	8,6
23	25,7	9,4	68,0	1,74	22,5	7,9
24	20,3	10,5	83,4	1,88	23,6	8,0
25	21,4	10,5	79,3	1,83	23,7	5,5
26	22,5	8,4	74,2	1,78	23,4	4,9
27	24,6	11,5	78,3	1,83	23,4	7,4
28	23,5	13,6	66,0	1,72	22,3	7,7
29	22,5	10,5	68,0	1,71	23,3	5,9
30	22,5	11,5	71,1	1,77	22,7	6,7
31	22,5	10,5	76,3	1,80	23,5	7,7
32	22,5	10,5	77,3	1,80	23,9	7,1
33	22,5	9,4	75,2	1,82	22,7	7,9
34	19,3	8,4	74,2	1,78	23,4	7,0

VO_{2max} (ml/kg/min)	maxHR (beats/min)	YO-YO IR2 (m)	PR 1 -Mean HR (b/min)	PR 2 -Mean HR (b/min)
52,5	198,0	1267	124	145
57,5	188,9	1505	126	141
59,3	204,0	1228	122	154
65,4	207,0	1069	146	139
53,3	196,0	1426	125	133
62,7	210,0	1149	136	138
63,6	199,0	1307	129	152
64,9	200,0	1149	143	146
49,6	192,0	1267	128	139
66,3	206,0	1307	127	147
61,0	189,0	1545	127	138
56,3	195,0	1624	137	133
56,3	201,0	1545	129	135
61,8	198,0	1347	125	142
66,6	193,0	1228	133	147
55,9	200,0	1307	131	149
59,4	202,0	1465	124	140
69,6	191,0	1228	126	141
54,9	206,0	1267	128	138
53,5	202,0	1386	123	145
56,5	197	1465	128	146
62,6	197	1624	133	135
60,9	196	1584	121	141
55,6	195	1505	124	137
66,9	194	1505	122	153
68,5	203	1426	115	151
56,9	200	1347	130	132
57,9	195	1584	127	138
61,1	201	1663	134	153
54,5	202	1149	125	141
55,0	196	1149	138	134
57,2	193	1386	119	132
54,3	202	1030	114	137
56,3	203	1109	123	135

PR 1 -Max HR (b/min)	PR 2 -Max HR (b/min)	PR 1 - total distance (m)	PR 2 - total distance (m)
171	186	3202,6	6151,6
181	193	2986,7	6202,3
184	190	3145,1	6045,7
177	184	3363,8	6039,6
165	181	2896,2	5971,8
182	189	3192,7	6211
169	180	3077,2	6089,7
178	192	3086,6	6046,2
192	194	3132,4	5951,3
187	191	3240,5	6146,4
173	183	3284,1	6181,5
164	179	3215,9	6096,7
173	184	3186,7	6023,5
170	186	3251,6	6204,7
175	184	3014,7	6008,9
177	190	3077,2	5994,1
182	193	3236,4	6151,8
169	179	3181,8	6230,5
173	185	3206,6	6066,1
183	188	3114,3	6099,3
180	186	3311,6	6281,2
186	189	3159,8	6195,5
175	182	3286,2	6305,5
171	186	3312	6259
169	175	3421,3	6149,7
179	185	2986,1	6192,3
183	189	3944,8	6411,8
192	193	3071,2	6311,2
189	196	3136,9	6061,6
171	185	3059,8	6133,7
174	189	3275,3	6258,9
181	185	3360,4	6241,5
183	196	3226,5	6259,3
183	198	3304,1	6226,1

PR 1 - distance at 0-7.2 km/h (m)	PR 2 - distance at 0-7.2 km/h (m)	PR 1 - distance at 7.3-14.4 km/h (m)
2358,1	3248,3	586,7
2138,9	3372,7	592,3
2280,9	3145,5	606,4
2489,5	3112,8	612
2057,3	3168,2	587,6
2346,1	3458,3	589,2
2220,6	3070	604,1
2242,2	3255,2	613,4
2293	3107,7	602,7
2333,5	3233,4	629,5
2424	3314,8	595,9
2358,6	3310	613,2
2345,4	3157,4	593
2401	3279,6	596,4
2178,5	3230	591,8
2234,3	3088,7	597
2381,9	3259,5	599,8
2358	3356,4	567
2348,5	3217,4	604,6
2254	3228,6	606,9
2495,7	3337,7	559,1
2352,1	3172,8	549,3
2462,2	3155,8	550,6
2508,8	2842,5	545,2
2606,5	3153,7	561,6
2168,8	3227,1	563,4
3139,2	3436,8	552,8
2255,1	3290,7	555
2309,4	3116,8	559,5
2229,3	3028,7	564,1
2446,8	3174,9	560,9
2527,5	3188,9	558
2403,7	3219,5	556,1
2481,4	3171,1	557,6

PRACTICE DATA

PR 2 - distance at 7.3-14.4 km/h (m)	PR 1 - distance at 14.5-19.8 km/h (m)
1915,8	185,1
1837,1	184,2
1936,2	183,9
1945,1	190,7
1850,3	180,5
1786,9	188
2035,4	177,1
1806,7	169,4
1864,6	177,3
1921,7	192,9
1914,3	185,6
1825	182,4
1891,9	181,7
1941,5	188,1
1795,4	176,3
1930,8	179,8
1917,7	184,4
1899,6	186
1874,1	182,3
1901,2	182,9
1932,6	164
2016,8	161,4
2159,6	169,7
2412,3	162,3
1998,1	160,8
1975,6	163,6
1983,5	157,9
2012	161,5
1949,3	162,6
2111,4	164
2092,7	166,7
2057,1	169,8
2046,2	165,2
2056,9	163

PR 2 - distance at 14.5-19.8 km/h (m)	PR 1 - distance at 19,8-25.2 km/h (m)
674,5	52,1
681,2	49,4
662,8	51,6
669,4	47
649,7	45,8
659,6	46,1
672	55,6
679,1	40,9
675,3	38,2
688	61,9
651,9	53,7
662,7	41,2
671,4	43,8
679,5	45,5
677	44,7
675,1	42
670,5	45,1
673,2	47,3
669,6	48,1
668,3	46,9
629,4	66,5
625,1	69,2
623,4	70,6
630,2	63,1
617,9	62,9
619,6	65,7
621	69,1
626,7	71,6
620,8	70,3
622,1	69
625,7	70,9
621,9	73,5
622,5	71,2
620,7	70,1

PR 2 - distance at 19,8-25.2 km/h (m)	PR 1 - distance at >25.2 km/h (m)	PR 2 - distance at >25.2 km/h (m)
225,1	20,6	87,9
223,2	21,9	88,1
220,6	22,3	80,6
229,8	24,6	82,5
219,7	25	83,9
221,5	23,3	84,7
226,3	19,8	86
224	20,7	81,2
222,8	21,2	80,9
220,9	22,7	82,4
216,9	24,9	83,6
218,5	20,5	80,5
223,4	22,8	79,4
220,6	20,6	83,5
218,4	23,4	88,1
217,5	24,1	82
220,2	25,2	83,9
219,7	23,5	81,6
221	23,1	84
216,9	23,6	84,3
246,3	26,3	135,2
250,4	27,8	130,4
239,1	33,1	127,6
244,8	32,6	129,2
249,7	29,5	130,3
243,6	24,6	126,4
242,8	25,8	127,7
252,5	28	129,3
244	35,1	130,7
240,2	33,4	131,3
238,6	30	127
244,1	31,6	129,5
245,2	30,3	125,9
245,2	32	132,2

GAME DATA				
Resting HR (b/min)	Mean HR (b/min)	Peak HR (b/min)	Resting Lactate (mM)	Post-game Lactate (mM)
64	165,0	195	0,93	4,83
65	168,0	194	0,99	5,49
66	158,0	188	1,21	5,68
63	165,0	185	1,5	6,09
69	170,0	199	1,14	3,38
68	162,0	182	1,04	5,87
59	173,0	202	1,03	4,15
61	149,0	177	0,79	4,77
69	167,0	194	1,62	6,87
62	170,0	197	1,27	7,63
72	164,0	193	1,3	4,72
70	154,0	194	1,11	4,79
64	176,0	201	1,22	5,24
65	171,0	195	1,17	3,98
63	145,0	196	1,12	4,86
78	170,0	197	1,06	4,51
66	174,0	202	1,25	4,04
67	165,0	191	0,98	4,13
64	161,0	196	1,11	4,88
67	172,0	198	1,08	4,68
67			1,38	
65			1,23	
83			1,01	
76			0,83	
68			1,51	
57			1,27	
72			1,35	
71			1,18	
63			1,1	
69			0,95	
68			1,26	
60			1,47	
67			1,3	
70			1,38	

Total distance (m)	Distance at 0,2-7,2 km/h (m)	Distance at 7,3-14,4 km/h (m)
10092	3271,4	4127,6
10281	3318,4	4204,9
8950	2856,0	3660,6
8720	2821,0	3566,4
11438	3217,3	4678,1
8925	2897,5	3650,3
11844	3849,4	4844,2
9543	3108,0	3903,1
10944	3581,2	4476,1
10992	3537,7	4495,7
10152	3255,4	4152,2
10255	3300,8	4194,3
12461	4005,6	5096,5
10294	3282,7	4210,2
8878	2859,3	3631,1
11418	3729,9	4670,0
12074	3905,1	4938,3
9715	3173,0	3973,4
9713	3130,9	3972,6
11297	3681,3	4620,5

Distance at 14,5-19,8 km/h (m)	Distance at 19,9-25,2 km/h (m)	Distance at > 25,2 km/h (m)
1856,9	600,0	236,0
1891,7	607,0	259,0
1646,8	543,0	243,6
1604,4	519,1	209,0
2104,6	686,0	252,0
1642,2	536,0	199,0
2179,3	700,0	271,1
1755,9	558,0	218,0
2013,7	647,0	226,0
2022,5	659,0	277,0
1868,0	604,4	272,0
1886,9	606,0	267,0
2292,8	752,0	314,0
1894,1	623,0	284,0
1633,6	524,0	230,0
2100,9	675,0	242,2
2221,6	714,0	295,0
1787,6	573,0	208,0
1787,2	578,3	244,0
2078,6	672,6	244,0

GAME ACTIVITY PROFILE (GPS DATA)

Acceleration distance (m) at 1–2 m/s^2	Acceleration distance (m) at (2-3 m/s^2)
654	170,4
619	197,9
693	161,5
781	148,7
513	280,6
814	144,8
541	244,2
793	152,6
627	162,5
539	261,9
549	226,5
517	221,6
498	298,4
588	203,8
725	141,8
563	209,7
502	269,8
742	159,5
714	186,1
528	237,3

Acceleration distance (m) at (> 3 m/s^2)	Decceleration distance (m) at 1–2 m/s^2
136,1	564,1
161,5	536,6
90,7	582,1
70,8	643,1
215,8	446,3
68,6	691,1
198,1	513,5
105,1	659,1
154,9	603,1
211,3	477,8
178,1	469,4
162,7	485,2
236,8	433,7
188,1	496,7
74,1	625,1
169,3	551,3
225,7	431,6
123,9	612,1
146,1	596,1
201,4	522,3

Decceleration distance (m) at (2-3 m/s^2)	Decceleration distance (m) at (> 3 m/s^2)
165,5	118,6
205,0	146,0
150,8	106,5
129,1	88,2
210,9	183,5
138,0	91,3
192,2	175,4
140,9	95,3
167,5	122,7
249,3	172,4
181,3	136,9
198,1	153,1
187,2	204,8
241,4	131,8
120,2	86,2
176,4	161,2
235,5	193,7
148,8	99,4
156,7	113,6
224,7	198,7
