# Supplementary material for: Recovery Kinetics of Knee Flexor and Extensor Strength after a Football Match
Source: PLoS One. 2015 Jun 4;10(6):e0128072. doi: 10.1371/journal.pone.0128072 (PMC4456353; doi:10.1371/journal.pone.0128072)
Supplement: S2 File — (PDF) [file pone.0128072.s002.pdf]

	DOMS KNEE FLEXORS - DOMINANT LIMB					DOMS KNEE FLE	
SUBJECT ID	START-PREP	G1-POST	G1-1D POST	G1-2D POST	G1-3D POST	START-PREP	G1-POST
1	1	4,0	6,4	5,4	3,0	1	2,5
2	1	4,6	7,7	4,5	2,8	1	2,7
3	1	5,0	7,9	6,4	2,4	1	3,3
4	1	4,4	7,1	5,6	3,4	1	2,9
5	1	4,0	6,6	4,9	2,8	1	2,6
6	1	4,2	7,5	4,1	2,4	1	3,3
7	1	4,6	8,1	5,2	1,7	1	4,5
8	1	4,8	7,5	3,7	2,8	1	4,3
9	1	5,0	6,2	5,2	3,2	1	3,7
10	1	4,6	6,4	3,9	2,1	1	3,1
11	1	5,5	8,1	4,5	1,7	1	4,1
12	1	5,7	7,3	5,2	1,5	1	3,9
13	1	4,8	7,9	4,5	1,7	1	3,3
14	1	4,6	7,7	4,1	1,9	1	3,7
15	1	5,0	7,7	4,3	1,1	1	4,1
16	1	4,4	6,6	4,9	3,4	1	3,9
17	1	4,8	8,6	5,4	3,0	1	3,7
18	1	5,3	7,9	3,5	2,6	1	4,7
19	1	4,8	6,8	6,2	3,8	1	3,9
20	1	4,6	6,2	6,2	4,1	1	3,9
21	1	1,0	1,0	1,0	1,6	1	1
22	1	1,0	1,0	1,0	1,6	1	1
23	1	1,0	1,0	2,0	1,8	1	1
24	1	1,0	1,0	1,0	1,6	1	1
25	1	1,0	1,0	1,0	1,6	1	1
26	1	1,0	1,0	1,0	1,4	1	1
27	1	1,0	1,0	2,0	1,4	1	1
28	1	1,0	2,0	1,0	1,2	1	1
29	1	1,0	1,0	1,0	1,6	1	1
30	1	1,0	1,0	1,0	1,4	1	1
31	1	1,0	1,0	2,0	1,2	1	1
32	1	1,0	1,0	2,0	1,4	1	1
33	1	1,0	1,0	1,0	2,0	1	1
34	1	1,0	1,0	2,0	1,6	1	1

MUSCLE DAMAGE MARKERS

EXORS/ NON-DOMINANT LIMB			DOMS KNEE EXTENSORS / DOMINANT LIMB				
G1-1D POST	G1-2D POST	G1-3D POST	START-PREP	G1-POST	G1-1D POST	G1-2D POST	G1-3D POST
5,4	4,4	2,3	1	3,9	6,0	5,1	2,9
4,8	4,2	2,7	1	3,7	5,6	5,5	2,6
5,0	3,8	1,7	1	4,5	6,9	5,3	2,0
5,2	4,0	2,3	1	3,9	6,3	5,1	2,7
4,8	4,0	2,9	1	3,3	6,0	5,5	3,3
6,1	3,2	1,8	1	4,7	6,3	3,9	1,8
6,3	3,4	1,2	1	4,9	7,3	3,7	1,6
6,7	3,6	1,5	1	4,5	7,1	3,9	1,4
6,1	4,4	2,1	1	3,7	5,8	5,7	2,7
6,3	3,6	1,9	1	4,1	6,3	4,5	2,0
6,5	2,7	1,1	1	5,5	7,5	3,1	1,6
6,9	3,2	1,5	1	5,1	6,7	3,3	1,8
7,3	3,4	1,7	1	4,9	6,9	3,7	1,8
5,6	2,9	2,3	1	4,5	6,5	3,9	1,4
6,1	3,4	1,9	1	4,7	7,7	3,1	1,6
5,4	2,0	2,5	1	3,9	6,0	4,9	2,4
6,3	2,9	1,7	1	4,3	6,7	3,9	1,4
6,5	2,5	1,5	1	4,5	7,3	3,7	1,6
5,0	3,8	2,5	1	3,3	6,0	4,7	2,4
5,4	4,2	2,3	1	3,5	6,3	5,1	2,7
1	1,0	1,1	1	1,0	1,0	1,0	2,0
1	1,0	1,3	1	1,0	1,0	1,0	1,6
1	1,0	1,3	1	1,0	1,0	1,0	1,4
1	1,0	1,5	1	1,0	1,0	2,0	1,6
1	1,0	1,7	1	1,0	1,0	1,0	1,4
1	1,0	1,3	1	1,0	1,0	1,0	1,4
1	2,0	1,5	1	1,0	1,0	2,0	1,6
1	1,0	1,3	1	1,0	1,0	2,0	1,6
1	1,0	1,5	1	1,0	1,0	1,0	1,4
1	2,0	1,5	1	1,0	1,0	2,0	1,4
1	2,0	1,3	1	1,0	1,0	1,0	1,6
1	1,0	1,1	1	1,0	1,0	1,0	1,8
1	1,0	1,3	1	1,0	1,0	1,0	1,2
1	1,0	1,1	1	1,0	1,0	1,0	1,2

					INFLA			
DOMS KNEE EXTENSORS / NON-DOMINANT LIMB					CREATINE KINASE ACTIVITY (U/L)			
START-PREP	G1-POST	G1-1D POST	G1-2D POST	G1-3D POST	START-PREP	G1-POST	G1-1D POST	G1-2D POST
1	2,5	4,3	3,7	2,2	119,8	468,5	579,9	771,3
1	2,7	4,5	3,5	2,0	132,8	381,3	513,6	724,1
1	3,5	5,1	2,7	1,6	172,6	537,2	867,2	1370,1
1	2,9	4,9	3,3	2,0	148,9	463,1	687,9	983,7
1	2,7	4,7	3,3	2,6	109,0	333,4	439,1	592,8
1	2,9	5,3	2,9	1,6	100,9	288,8	397,5	540,7
1	3,3	5,7	2,6	1,2	204,8	631,6	950,7	1426,1
1	3,1	5,5	2,9	1,4	145,4	362,5	473,8	663,4
1	2,5	4,7	3,3	2,2	119,1	383,8	486,5	627,6
1	2,9	4,7	2,9	1,8	131,3	340,0	437,7	590,9
1	4,1	6,0	2,2	1,0	184,4	567,8	955,8	1452,9
1	3,5	5,3	2,4	1,2	249,4	875,4	1213,7	1577,8
1	3,5	4,9	2,7	1,4	192,5	629,6	1122,3	1627,3
1	3,3	5,5	2,6	1,4	188,6	523,3	829,2	1235,5
1	3,7	6,2	2,6	1,2	168,8	449,8	739,4	1146,1
1	2,7	4,3	3,1	1,8	172,8	580,6	845,1	1174,7
1	3,3	4,9	2,7	1,6	204,3	616,3	952,1	1399,5
1	3,3	5,1	2,6	1,0	156,8	427,8	804,9	1287,8
1	2,7	4,3	3,3	2,0	151,4	489,8	649,9	909,9
1	2,7	4,1	3,7	2,2	160,0	525,7	681,9	941,1
1	1,0	1,0	1,0	1,4	151,0	166,8	256,1	265,7
1	1,0	1,0	1,0	1,4	164,6	171,0	274,0	293,0
1	1,0	1,0	1,0	1,4	246,5	203,8	296,9	341,9
1	1,0	1,0	2,0	1,4	82,4	88,1	150,9	163,9
1	1,0	1,0	1,0	1,6	127,1	93,9	138,0	170,3
1	1,0	1,0	2,0	1,4	150,8	177,8	244,7	270,3
1	1,0	1,0	1,0	1,4	97,8	101,4	157,8	195,2
1	1,0	1,0	2,0	1,2	155,0	171,7	255,6	282,0
1	1,0	1,0	1,0	1,0	193,5	194,3	280,7	301,6
1	1,0	1,0	1,0	1,4	202,7	162,7	237,7	272,5
1	1,0	1,0	1,0	1,2	159,5	142,7	213,8	249,2
1	1,0	1,0	1,0	1,2	227,3	201,0	282,9	328,7
1	1,0	1,0	2,0	1,2	143,9	153,0	215,4	232,3
1	1,0	1,0	1,0	1,4	240,1	169,9	238,2	273,7

MMATORY MARKERS					ISOMETRIC TORQUE OF K			
White Blood Cell Count (KX10^3)					DOMINANT LEG			
START-PREP	G1-POST	G1-1D POST	G1-2D POST	G1-3D POST	PRE	1D POST	2D POST	3D POST
6,8	12,3	8,1	7,2	7,2	247,3	217,1	232,2	229,2
7,2	14,9	9,0	7,1	7,3	287,0	255,4	261,4	275,2
7,6	13,3	8,4	7,5	7,5	259,9	236,2	245,0	247,9
6,1	12,6	8,0	6,3	6,1	237,0	227,3	225,8	225,7
6,4	12,0	7,5	6,7	7,0	243,2	215,0	232,4	232,3
6,9	13,4	8,1	6,6	7,2	294,5	285,1	284,3	281,8
6,3	11,9	8,0	6,9	6,8	303,8	227,8	291,0	294,9
6,0	12,8	7,7	6,5	6,6	307,4	296,6	299,0	297,5
6,7	13,2	8,4	6,3	6,7	251,8	232,9	235,8	244,0
7,1	14,1	8,6	6,8	7,1	226,0	204,1	211,2	214,0
7,2	13,8	9,1	7,7	7,1	287,4	263,0	268,1	276,5
7,8	12,4	7,5	7,2	7,7	286,7	272,4	275,8	270,4
6,6	11,8	7,6	6,9	6,5	268,9	243,3	254,9	259,5
7,4	14,6	8,8	7,8	7,7	251,8	237,5	234,2	236,2
7,2	13,1	9,3	8,0	7,8	229,4	223,0	209,6	214,3
7,3	13,3	8,2	7,7	7,6	216,4	202,1	208,5	209,9
6,8	13,2	8,5	7,9	7,5	288,2	264,2	272,5	280,2
7,1	11,8	7,8	7,6	7,8	278,1	261,1	257,3	258,6
7,9	16,4	9,6	8,4	8,0	212,9	197,6	195,3	202,3
7,5	14,5	8,6	8,0	7,8	294,9	271,3	271,3	284,9
6,8	6,9	6,9	7,2	7,2	257,3	255,2	260,3	256,0
6,4	6,7	6,8	6,9	7,0	294,1	291,2	297,3	290,4
6,0	6,3	6,6	6,6	6,8	367,2	267,8	269,2	263,1
7,7	7,5	7,3	7,4	7,2	203,5	292,8	291,2	285,3
7,1	7,6	7,2	7,4	7,5	287,8	293,4	287,1	283,9
5,8	6,4	6,6	6,9	6,9	244,1	245,5	248,4	241,0
8,1	7,3	7,5	7,2	6,9	281,1	286,5	284,2	282,6
6,9	7,1	7,3	7,1	7,3	222,7	223,5	218,2	226,1
6,4	7,0	7,4	7,6	7,8	255	259,7	252,2	255,6
7,4	7,2	6,9	6,8	6,9	235,3	236,0	240,2	232,4
6,6	7,0	6,7	6,9	7,1	242,3	244,7	239,3	247,0
6,2	6,5	6,8	6,5	6,5	263,6	265,7	264,2	263,4
7,0	6,5	6,6	7,0	6,8	297,8	302,3	299,3	294,0
6,3	6,6	6,9	7,2	7,4	249,3	250,7	245,5	247,0

ISOMETRIC STRENGTH DATA								
NEE EXTENSORS (Nm/kg)				ISOMETRIC TORQUE OF KNEE FLEXO				
NON-DOMINANT LEG				DOMINANT LEG				
PRE	1D POST	2D POST	3D POST	PRE	1D POST	2D POST	3D POST	PRE
245,1	225,3	234,4	238,5	131,2	117,4	117,3	120,1	129,8
280,8	266,7	267,6	266,7	154,3	135,5	141,1	145,9	150,5
262,0	244,7	246,3	250,8	152,2	139,3	139,6	139,9	148,6
243,1	227,8	230,7	233,2	125,0	115,6	112,4	115,5	123,5
238,1	221,9	227,3	232,6	132,0	118,4	124,1	127,1	133,5
286,3	261,7	267,4	275,7	163,6	153,0	150,5	154,9	159,9
305,3	277,9	296,5	301,7	178,9	159,2	171,6	165,9	174,1
303,8	282,5	291,1	288,0	172,0	160,3	158,5	159,9	168,4
249,5	234,5	237,3	241,0	136,7	123,9	123,6	129,8	138,3
222,6	206,6	210,4	212,0	113,7	98,9	108,2	108,0	117,1
289,0	276,3	277,5	279,8	166,4	150,3	154,1	156,4	164,0
286,7	263,8	270,1	273,8	150,4	137,2	139,0	144,0	147,4
272,9	262,0	264,5	267,5	145,8	128,6	138,1	141,3	147,5
248,9	237,2	239,4	244,4	137,4	118,8	127,5	128,0	135,1
226,6	205,5	210,5	213,7	125,5	116,6	113,8	117,2	126,9
218,5	206,0	214,1	215,0	119,5	104,5	111,8	113,8	121,6
291,5	281,3	281,9	277,5	155,6	138,9	146,9	145,6	157,2
275,7	255,1	261,1	263,3	150,3	136,4	136,7	143,5	147,2
212,3	191,9	196,4	199,4	123,6	111,2	112,8	116,0	125,4
290,1	274,4	279,9	281,4	171,5	151,8	159,5	161,5	169,1
256,7	261,8	258,1	254,5	138,9	137,5	136,0	140,4	139,7
291,9	288,9	293,4	288,9	157,0	159,3	153,2	154,7	153,2
235,9	263,8	261,7	259,0	126,4	127,8	129,8	128,5	127,8
285,3	282,8	279,5	286,2	162,7	167,7	161,0	165,2	160,2
285,5	283,1	287,9	290,3	138,8	132,4	136,4	134,0	139,6
246,2	244,0	241,8	247,0	156,5	152,8	155,0	160,2	152,0
279,5	278,7	274,8	281,0	145,6	148,7	143,3	146,4	147,9
224,8	232,0	222,8	227,4	127,2	130,5	125,2	130,5	125,9
252,2	249,5	248,1	250,8	114,9	112,8	116,9	110,8	118,3
232,4	233,1	233,8	236,7	124,4	125,8	127,2	128,6	122,2
248,5	250,8	252,3	251,5	158,5	154,0	156,3	155,5	160,1
262,7	268,8	261,9	265,0	170,7	166,9	168,4	166,1	168,4
294,7	297,8	291,0	292,5	148,9	144,4	145,9	142,1	146,6
252,9	258,1	250,7	251,4	141,6	137,2	139,4	137,9	139,4

ɔRS (Nm/kg)			CONCENTRIC TORQUE OF KNEE EXTENSORS (Nm/s)				CONCENTRIC TORQUE O	
NON-DOMINANT LEG			DOMINANT LEG				DOMIN	
**1D POST**	**2D POST**	**3D POST**	**PRE**	**1D POST**	**2D POST**	**3D POST**	**PRE**	**1D POST**
118,3	122,0	125,2	217,7	199,3	208,8	211,6	124,0	111,5
139,0	141,7	145,6	246,6	226,5	243,4	242,9	144,3	130,1
137,5	140,4	142,2	227,6	211,0	221,0	221,4	141,4	128,4
116,3	115,7	117,9	213,4	198,5	200,0	208,1	122,7	114,1
120,8	124,7	129,5	218,0	192,1	203,3	215,2	122,4	105,8
150,3	152,5	152,2	249,1	233,3	234,8	239,9	153,2	140,9
155,5	167,0	165,4	261,6	232,3	242,9	256,6	170,2	144,8
157,7	156,6	163,5	271,7	250,3	251,4	266,0	162,2	148,7
128,1	132,7	134,5	227,9	207,6	218,6	220,1	132,1	118,1
107,0	108,5	115,2	198,3	177,6	185,2	192,3	105,6	94,0
147,1	157,8	159,9	250,8	226,4	239,3	246,5	156,1	138,3
136,9	142,1	143,9	249,5	226,1	241,3	247,0	145,2	127,4
133,7	142,3	144,1	238,7	211,0	228,6	237,0	137,7	119,8
123,9	130,8	129,6	222,3	198,3	207,5	217,2	134,4	120,7
118,4	117,1	120,3	200,1	189,0	191,4	192,3	123,4	113,8
111,8	117,8	118,9	193,7	169,9	178,2	193,5	112,0	98,8
146,3	149,7	150,9	247,3	222,6	234,8	243,3	144,1	123,8
135,6	139,7	141,4	241,7	221,7	226,6	232,5	147,2	134,2
113,0	115,1	118,4	186,0	172,0	180,2	180,0	119,9	108,5
153,8	160,1	162,8	259,6	235,1	249,3	258,6	162,7	142,0
136,8	135,3	138,2	226,0	226,0	224,5	223,8	132,4	132,4
150,2	148,6	155,5	251,5	253,1	247,7	248,5	150,9	150,2
129,2	125,8	126,4	230,5	230,5	229,1	227,7	125,1	125,1
156,8	162,7	156,0	249,5	251,1	248,6	247,0	156,8	154,3
138,8	143,6	142,0	251,4	253,0	249,8	249,8	136,4	137,2
154,3	155,7	149,1	215,1	218,0	215,8	212,8	149,8	149,1
154,2	147,9	149,5	249,7	250,5	248,9	247,4	142,5	140,9
122,6	125,2	121,3	197,8	202,4	200,4	196,4	122,6	120,0
116,2	121,7	119,0	225,7	223,0	220,9	219,6	114,2	116,2
120,8	118,7	118,7	209,7	211,8	208,2	206,1	120,8	122,2
156,3	154,7	159,3	215,7	217,2	213,4	211,1	152,4	154,7
164,5	166,1	162,2	231,0	233,3	230,2	228,7	156,0	153,0
142,9	143,6	140,6	257,9	256,4	255,6	254,1	145,1	145,1
135,0	137,2	135,7	219,5	216,5	213,6	212,1	136,5	137,9

								STRE
F KNEE FLEXORS (Nm/s)		ECCENTRIC TORQUE OF KNEE EXTENSORS (Nm/s)				ECCENTRIC TORQUE OF KNEE FLEXORS		
ANT LEG		DOMINANT LEG				DOMINANT LEG		
2D POST	3D POST	PRE	1D POST	2D POST	3D POST	PRE	1D POST	2D POST
111,7	120,3	243,7	229,0	237,1	237,1	142,0	121,7	126,7
139,9	141,5	273,8	246,9	269,7	271,0	166,8	139,3	151,7
127,1	134,6	259,2	245,3	248,8	252,9	166,6	145,2	145,9
108,5	115,1	238,6	227,2	226,4	229,5	137,2	121,7	118,1
117,5	121,1	246,2	226,1	244,2	245,0	132,0	109,9	123,0
136,3	143,8	284,1	273,3	270,4	274,7	166,6	149,6	142,2
156,2	164,2	295,8	261,9	286,9	290,5	178,1	146,2	157,2
145,0	154,9	309,2	293,6	292,5	298,0	188,0	161,5	161,1
120,2	128,4	257,2	237,5	248,0	251,6	156,8	132,2	138,7
98,0	99,8	227,4	202,0	222,4	224,4	121,1	96,7	108,7
149,0	152,0	290,6	265,2	286,8	290,3	166,4	137,2	150,2
139,8	144,1	279,3	263,3	277,9	277,3	157,1	132,8	143,5
129,8	135,9	270,5	241,3	265,1	269,4	149,9	118,9	138,8
125,3	132,3	250,4	223,9	247,4	247,4	149,9	122,0	137,7
109,6	118,6	225,9	216,5	215,8	218,7	130,4	112,4	112,8
105,2	109,4	221,2	198,7	216,1	219,2	123,0	97,1	109,3
132,9	143,5	280,0	246,9	278,4	275,6	161,3	130,2	151,7
133,2	141,1	281,2	262,6	271,9	272,7	155,7	132,6	136,8
109,6	115,5	209,2	194,4	200,5	205,3	135,2	114,9	117,8
154,4	159,4	296,5	269,1	291,2	295,9	175,5	147,3	159,3
130,9	129,4	253,0	253,8	250,8	250,1	142,6	142,6	139,7
148,6	148,9	291,9	292,7	289,6	287,3	166,2	166,9	163,9
124,4	123,7	262,4	262,4	260,4	259,0	141,4	142,1	140,7
155,2	152,7	288,7	287,8	283,7	282,0	166,0	166,9	164,4
135,6	134,8	289,5	290,3	285,5	281,6	152,3	150,7	146,7
148,3	146,8	245,5	248,4	244,0	240,3	171,3	172,8	169,8
141,4	138,6	283,4	285,7	283,4	281,8	152,6	152,6	151,1
120,0	118,7	222,8	224,1	223,5	221,5	133,8	135,1	131,8
116,9	114,2	252,9	254,2	250,2	248,1	126,4	124,4	122,4
120,8	121,5	236,7	238,8	238,8	235,2	136,5	135,0	134,3
153,2	153,2	240,9	240,9	238,6	238,6	164,6	163,1	160,1
151,4	149,9	259,6	258,8	257,2	254,9	173,0	173,8	167,6
144,4	142,9	293,2	292,5	291,0	290,2	166,9	168,4	163,9
137,2	138,7	251,4	253,6	250,7	251,4	152,8	152,0	149,1

NGTH DATA AT 60 DEG/S

(Nm/s)	CONCENTRIC TORQUE OF KNEE EXTENSORS (Nm/s)				CONCENTRIC TORQUE OF KNEE FLEXORS (Nm/s)			
	NON-DOMINANT LEG				NON-DOMINANT LEG			
**3D POST**	**PRE**	**1D POST**	**2D POST**	**3D POST**	**PRE**	**1D POST**	**2D POST**	**3D POST**
133,4	216,3	198,5	207,6	212,7	122,6	112,0	114,4	116,1
160,7	244,3	228,1	239,9	245,9	141,9	127,2	139,1	142,7
154,7	225,4	209,0	212,3	217,5	137,1	126,4	123,5	124,9
125,2	205,8	199,0	194,4	198,2	122,0	114,7	108,7	110,5
129,7	215,8	189,3	209,8	213,6	120,1	106,8	115,9	118,7
152,7	247,6	235,7	229,1	237,2	149,5	139,8	134,1	141,3
168,2	258,4	230,0	246,8	255,2	167,0	150,3	156,5	164,6
173,7	269,1	251,6	248,3	258,4	160,4	150,3	145,5	153,2
146,1	226,3	205,4	214,6	221,7	134,4	121,8	128,5	131,3
113,2	194,9	178,7	190,4	192,9	103,5	90,2	95,8	102,2
164,3	247,6	222,1	244,4	250,0	152,9	138,3	148,3	152,1
153,8	248,7	224,1	241,3	246,5	141,5	126,3	133,7	143,0
141,3	236,3	212,7	230,4	235,5	139,3	123,2	132,5	137,7
145,1	149,9	126,3	147,5	152,1	132,2	121,1	127,0	130,0
120,8	195,9	186,8	183,2	191,1	118,5	110,2	108,7	111,6
117,2	195,1	168,0	188,7	192,4	113,4	103,2	110,6	114,0
152,8	241,6	223,3	232,9	239,1	138,4	121,4	131,2	135,9
143,4	243,2	219,5	226,2	240,9	141,0	130,7	129,8	135,5
126,6	189,1	172,2	179,8	186,6	118,1	109,2	111,2	115,6
167,9	256,4	236,4	251,3	258,0	158,7	139,8	154,2	159,5
136,0	224,5	225,2	223,0	220,1	130,9	128,0	128,0	127,2
160,8	248,5	250,8	246,2	245,4	147,1	147,9	147,1	146,3
138,7	225,0	226,4	224,3	223,0	122,4	121,7	121,0	121,7
161,0	252,0	250,3	248,6	246,1	156,0	151,8	150,2	149,3
143,6	249,8	251,4	246,7	245,9	139,6	137,2	135,6	134,8
166,9	216,5	215,1	215,1	210,6	146,1	148,3	146,8	146,1
148,7	246,6	248,9	245,8	243,5	140,9	137,0	136,2	137,8
129,2	195,1	197,1	195,1	192,5	119,3	117,3	115,4	114,7
119,0	224,3	225,0	222,3	218,2	114,2	113,5	112,8	112,2
131,5	209,7	207,5	207,5	204,7	119,4	120,8	120,1	119,4
157,0	213,4	218,0	209,6	208,1	148,6	149,4	150,2	148,6
163,8	228,7	230,2	227,9	225,6	156,8	153,7	151,4	153,0
158,7	253,4	253,4	250,4	250,4	142,9	142,1	140,6	140,6
144,6	221,7	224,0	218,8	215,8	135,0	133,5	131,3	130,5

ECCENTRIC TORQUE OF KNEE EXTENSORS (Nm/s)				ECCENTRIC TORQUE OF KNEE FLEXORS (Nm/s)				CONCENTR
NON-DOMINANT LEG				NON-DOMINANT LEG				
PRE	1D POST	2D POST	3D POST	PRE	1D POST	2D POST	3D POST	PRE
240,1	222,5	229,3	233,9	138,4	119,5	124,6	131,5	134,6
272,2	254,2	259,0	264,6	163,6	140,4	153,0	155,0	177,9
254,9	241,5	252,0	252,3	165,1	143,2	144,6	155,7	164,0
236,3	226,5	234,0	233,9	138,0	121,5	121,5	129,3	155,9
246,2	226,3	241,0	240,3	129,8	110,2	121,9	123,9	163,5
276,6	267,0	268,5	274,4	160,6	140,1	145,7	151,8	158,8
292,6	265,8	281,5	288,8	179,7	150,8	166,4	166,9	208,5
310,9	299,0	306,5	301,3	185,3	161,2	168,6	173,1	177,8
254,9	232,5	248,0	247,0	156,8	136,3	144,7	152,0	172,6
228,1	214,7	219,9	223,3	121,8	104,6	111,1	114,4	146,5
290,6	270,6	280,3	283,6	160,8	139,1	152,6	158,4	207,2
272,6	257,6	263,6	267,1	159,4	134,7	148,0	155,1	193,8
268,0	245,4	261,5	263,2	146,7	125,1	138,1	143,1	163,4
245,2	234,4	232,6	241,3	148,4	127,8	136,3	145,5	158,4
224,5	217,3	221,0	218,9	129,0	112,9	115,7	119,3	155,8
221,9	204,6	213,0	219,9	120,9	103,4	109,3	118,7	134,4
278,4	264,9	272,7	272,3	156,4	133,1	145,8	155,0	169,1
277,3	264,6	272,6	269,8	157,2	136,6	144,0	148,1	163,1
208,0	195,2	201,3	204,9	130,3	111,9	115,6	125,1	130,6
289,3	261,4	280,5	279,4	173,1	148,2	154,6	167,0	172,7
251,6	250,8	249,4	248,6	140,4	139,7	138,9	137,5	151,4
291,2	291,9	289,6	289,9	164,6	166,9	163,9	163,9	170,7
263,1	265,1	261,7	262,4	140,7	139,4	140,0	138,7	159,1
287,0	284,5	283,7	285,3	163,5	163,5	161,0	160,2	189,4
286,3	288,7	289,5	287,9	150,7	148,3	147,5	146,7	193,5
244,7	246,2	244,7	245,5	170,6	171,3	169,1	167,6	140,2
281,0	279,5	275,5	274,0	152,6	151,9	151,9	148,7	159,7
223,5	222,8	220,8	222,2	131,2	129,9	128,5	129,2	148,3
251,5	250,2	250,2	248,1	125,1	126,4	124,4	123,0	176,1
238,1	235,2	233,8	233,1	135,0	135,7	135,0	131,5	152,8
237,0	236,3	234,8	251,5	160,1	161,6	159,3	157,0	168,4
255,7	254,2	252,6	238,7	164,5	163,8	162,2	159,1	163,8
289,5	288,0	285,7	286,5	164,7	163,2	161,7	160,9	193,2
249,9	249,9	247,7	247,7	154,3	155,0	155,7	152,0	144,6

RIC TORQUE OF KNEE EXTENSORS (Nm/s)			CONCENTRIC TORQUE OF KNEE FLEXORS (Nm/s)				ECCENTRIC TORQUE OF K	
DOMINANT LEG			DOMINANT LEG				DOMIN	
1D POST	2D POST	3D POST	PRE	1D POST	2D POST	3D POST	PRE	1D POST
123,3	127,6	130,5	96,6	77,9	85,8	88,7	251,6	222,8
160,5	171,4	173,7	108,6	83,8	99,3	103,2	287,0	259,0
151,5	156,5	160,1	112,0	94,0	96,9	104,1	269,2	247,7
144,8	147,9	151,7	93,0	78,5	80,0	83,8	250,8	234,8
147,6	160,2	162,4	92,7	71,2	83,1	89,0	252,9	218,8
148,0	151,7	154,7	119,7	102,6	104,1	112,3	293,7	275,2
190,8	202,8	204,4	136,8	108,1	122,5	128,8	308,5	283,1
166,6	172,8	174,6	127,4	109,6	108,7	115,8	322,5	299,4
158,4	162,2	163,8	101,2	82,7	91,2	98,1	265,0	233,3
134,0	139,4	144,8	77,1	62,3	67,7	70,4	235,5	214,5
189,5	202,2	203,8	123,4	94,0	113,9	119,4	295,4	253,2
176,5	186,9	192,9	107,2	84,9	96,8	101,3	286,0	261,4
149,1	160,5	162,9	111,6	87,2	104,3	106,7	281,9	250,9
145,5	153,6	156,6	99,7	82,0	88,6	94,5	256,3	229,7
143,6	145,7	151,3	94,8	82,3	83,0	87,9	233,6	220,3
123,0	129,2	131,9	92,7	75,6	81,8	85,9	230,8	201,3
153,1	163,0	167,9	108,1	81,1	96,6	105,6	290,7	262,9
151,0	155,7	161,1	110,8	92,9	94,5	99,9	295,1	275,0
120,5	126,6	127,9	102,2	85,0	89,9	93,6	214,7	198,2
154,7	163,5	170,7	125,8	97,0	114,6	119,4	306,9	276,5
152,8	151,4	149,2	92,9	91,4	91,4	87,8	258,9	260,3
168,4	166,2	168,4	117,4	118,9	117,4	117,4	301,1	301,8
156,3	153,6	132,6	95,2	95,9	93,8	92,5	267,2	269,9
193,6	191,9	186,9	126,0	126,8	125,1	124,3	296,2	297,0
192,7	189,6	190,3	110,2	114,2	106,3	104,7	300,6	303,0
144,6	146,1	138,7	101,6	100,1	98,6	98,6	247,7	250,7
161,3	157,3	158,1	107,2	106,5	103,3	101,8	285,7	286,5
152,9	149,6	144,4	96,9	94,9	93,6	89,7	232,0	232,0
167,9	167,2	174,0	84,3	85,0	82,3	80,2	267,8	265,8
154,9	152,8	149,2	95,9	98,1	93,1	91,7	233,8	234,5
171,5	167,7	165,4	122,0	122,7	119,7	117,4	253,1	254,6
168,4	170,0	160,7	128,2	132,9	125,9	120,5	263,4	265,7
191,7	188,7	190,2	106,0	109,0	105,3	101,5	302,3	305,3
149,1	146,8	143,1	97,9	100,9	96,4	94,2	261,0	263,3

STRENGTH DATA AT 180 DEG/S

KNEE EXTENSORS (Nm/s)		ECCENTRIC TORQUE OF KNEE FLEXORS (Nm/s)				CONCENTRIC TORQUE OF KNEE EXTENSO		
ANT LEG		DOMINANT LEG				NON-DOMINANT LEG		
2D POST	3D POST	PRE	1D POST	2D POST	3D POST	PRE	1D POST	2D POST
239,4	243,0	154,3	116,1	129,1	144,2	131,2	117,5	119,7
278,4	286,2	176,8	121,8	152,0	167,5	169,9	150,5	162,1
257,0	265,6	173,7	132,8	134,2	155,8	157,9	145,0	145,0
235,5	242,4	149,4	120,4	117,4	134,9	151,7	140,2	142,5
245,5	251,4	139,4	96,4	117,2	130,5	156,5	136,5	152,8
281,1	287,8	177,0	140,6	146,5	164,3	154,7	144,3	142,8
295,8	303,0	188,5	121,7	155,9	174,9	202,8	182,1	194,0
310,9	312,7	201,4	155,9	162,2	183,5	172,8	160,4	159,5
249,5	257,2	169,2	118,2	136,7	154,5	167,6	145,2	158,4
227,4	226,7	128,6	84,6	105,6	119,1	144,8	130,6	134,7
288,2	294,6	183,1	129,0	158,4	172,8	197,5	178,3	187,9
277,8	282,2	169,0	121,4	146,7	158,6	186,2	169,0	178,7
276,2	281,9	165,4	110,0	144,2	155,6	156,4	134,4	150,7
246,7	249,6	161,0	113,7	130,7	151,4	157,3	133,7	147,0
226,6	228,0	149,2	121,3	119,2	136,7	149,2	138,8	144,3
219,2	228,1	135,3	96,2	112,7	124,3	123,0	109,2	119,5
283,3	288,2	178,5	130,2	151,5	168,7	162,1	142,5	157,2
282,7	288,9	173,5	128,6	137,1	159,6	157,2	144,1	147,2
203,1	205,6	148,1	107,1	120,5	136,4	125,4	114,4	119,3
296,5	302,1	188,3	127,4	153,9	177,9	165,1	144,2	165,1
258,9	256,7	154,3	155,7	154,3	150,6	147,0	148,5	147,0
298,8	297,3	175,3	174,5	173,0	171,5	166,9	165,4	164,6
267,2	264,4	150,2	147,6	148,2	144,8	153,0	153,0	151,6
292,0	292,0	185,2	185,1	181,9	177,7	183,5	181,9	181,9
299,8	299,0	169,7	171,3	170,5	166,6	186,4	185,6	184,0
246,2	244,7	181,7	179,6	178,0	172,8	135,0	137,2	133,5
285,7	283,4	165,2	164,4	169,1	166,0	153,4	155,0	151,9
228,1	224,1	143,7	144,5	141,1	137,8	147,0	151,0	148,3
265,8	266,5	132,6	134,6	135,3	129,8	170,0	172,0	170,6
234,5	233,1	146,4	144,3	142,1	138,6	152,8	154,9	150,7
260,7	259,9	180,6	179,3	176,1	173,0	170,0	172,3	168,4
260,3	256,5	180,0	183,2	178,4	177,7	156,0	157,6	152,2
297,8	295,5	174,4	171,5	166,9	163,9	183,5	185,7	181,2
260,3	258,1	163,9	161,0	157,2	152,0	139,4	141,6	137,9

ORS (Nm/s)	CONCENTRIC TORQUE OF KNEE FLEXORS (Nm/s)				ECCENTRIC TORQUE OF KNEE EXTENSORS (Nm/s)			
	NON-DOMINANT LEG				NON-DOMINANT LEG			
3D POST	PRE	1D POST	2D POST	3D POST	PRE	1D POST	2D POST	3D POST
129,8	95,2	82,9	86,5	88,7	245,9	226,4	232,9	237,9
167,5	107,0	90,0	100,1	103,9	283,1	253,6	278,4	280,0
151,5	109,8	94,8	97,6	103,4	269,9	254,9	259,2	262,0
146,3	93,8	81,6	86,9	88,4	247,0	232,5	237,8	242,4
155,0	94,2	78,6	87,5	92,0	249,2	219,5	245,5	247,0
149,5	116,8	102,6	104,9	111,5	289,3	274,4	278,9	281,8
201,2	132,0	108,1	122,5	124,8	306,1	279,9	288,6	299,0
167,5	126,5	111,4	114,0	120,3	320,7	302,0	310,1	307,4
166,1	98,9	85,7	91,2	95,0	261,1	237,2	252,6	255,7
140,8	78,5	67,7	72,4	75,1	233,5	204,4	219,3	229,4
195,9	120,2	100,3	113,1	118,6	288,2	261,2	282,6	285,8
183,2	103,5	88,6	96,8	102,0	278,5	257,7	272,6	276,3
155,6	107,5	88,8	101,0	104,3	275,4	253,4	272,1	273,7
153,6	101,9	86,4	96,7	99,0	251,8	219,3	245,9	248,1
145,7	94,1	83,0	87,2	90,7	229,4	213,4	217,6	220,3
120,9	91,4	78,3	83,8	85,2	226,7	204,0	224,0	224,0
161,3	105,6	90,1	98,3	102,4	284,1	251,4	276,8	283,3
154,1	107,7	93,7	97,6	102,2	290,5	272,6	278,1	280,4
123,0	99,1	84,4	89,9	93,0	212,9	198,8	203,1	208,6
162,7	123,4	102,6	113,0	117,8	301,3	268,4	292,5	301,3
144,1	91,4	89,9	89,2	87,8	254,5	253,0	253,0	251,6
161,6	116,6	115,1	115,9	114,3	293,4	290,4	289,6	285,8
150,2	93,8	92,5	91,8	92,5	262,4	261,7	260,4	258,3
179,4	125,1	126,0	124,3	121,8	288,7	290,3	287,8	287,0
179,2	111,0	111,8	110,2	108,7	295,0	293,4	295,0	294,2
134,2	99,4	96,4	96,4	95,7	238,1	240,3	240,3	238,8
151,1	103,3	104,1	102,5	100,2	280,2	277,9	275,5	275,5
146,3	92,9	91,6	88,3	87,0	226,8	224,1	223,5	222,8
166,6	82,9	82,3	80,9	78,9	263,1	260,4	258,3	254,9
148,5	94,5	93,1	91,0	90,3	231,0	228,1	227,4	226,0
168,4	118,1	115,1	116,6	116,6	247,7	249,2	249,2	250,0
150,6	124,4	121,3	120,5	118,2	261,1	258,8	257,2	258,8
177,4	103,0	105,3	104,5	103,0	292,5	290,2	289,5	285,7
135,0	94,9	92,0	92,0	90,5	253,6	250,7	249,2	249,2

ECCENTRIC TORQUE OF KNEE FLEXORS (Nm/s)			
NON-DOMINANT LEG			
PRE	1D POST	2D POST	3D POST
144,9	108,9	124,0	131,9
167,5	121,0	148,1	160,5
168,7	134,2	139,3	157,9
143,3	122,7	117,4	131,1
129,8	94,2	115,7	124,6
168,8	133,1	142,0	155,4
182,9	128,8	163,8	174,1
193,3	149,7	163,9	181,8
158,4	115,9	135,2	146,0
120,5	90,0	104,2	116,4
176,8	125,0	154,5	168,0
163,1	132,6	146,7	155,6
155,6	110,8	139,3	147,5
151,4	115,9	132,2	146,2
140,2	116,5	122,0	129,7
124,3	94,1	104,4	117,5
168,7	121,2	145,8	163,8
164,2	125,5	144,8	154,9
140,1	104,0	113,8	130,3
178,7	132,2	153,9	166,7
152,8	152,1	149,2	144,1
172,3	170,0	167,7	163,1
143,4	142,8	139,4	134,6
173,5	176,0	175,2	162,7
159,4	157,0	153,9	153,1
170,6	167,6	163,9	163,9
155,8	154,2	152,6	144,8
135,8	135,1	133,2	130,5
120,3	120,3	116,9	133,2
136,5	135,0	132,2	127,9
171,5	168,4	166,9	162,3
173,0	172,3	170,7	171,5
164,7	166,2	163,9	157,1
146,1	144,6	144,6	135,7
